# Mass Spectrometry, Ion Mobility Separation and Molecular Modelling: A Powerful Combination for the Structural Characterisation of Substituted Cyclodextrins Mixtures

**DOI:** 10.3390/ijms232113352

**Published:** 2022-11-01

**Authors:** Sébastien Rigaud, Abdouramane Dosso, David Lesur, Dominique Cailleu, David Mathiron, Serge Pilard, Christine Cézard, Florence Djedaini-Pilard

**Affiliations:** 1 Laboratoire de Glycochimie des Antimicrobiens et des Agroressources, UMR CNRS 7378, Institut de Chimie de Picardie, FR CNRS 3085, F-80039 Amiens, France; 2 Plateforme-Analytique, Institut de Chimie de Picardie, FR CNRS 3085, Université de Picardie Jules Verne, F-80039 Amiens, France

**Keywords:** cyclodextrin isomers, MS/MS, IM-MS, NMR, molecular dynamics

## Abstract

When working on the synthesis of substituted cyclodextrins (CDs), the main challenge remains the analysis of the reaction media content. Our objective in this study was to fully characterise a complex isomers mixture of Lipidyl-βCDs (LipβCD) obtained with a degree of substitution 1 (DS = 1) from a one-step synthesis pathway. The benefit of tandem mass spectrometry (MS/MS) and ion mobility separation hyphenated with mass spectrometry (IM-MS) was investigated. The MS/MS fragment ion‘s relative intensities were analysed by principal component analysis (PCA) to discriminate isomers. The arrival time distribution (ATD) of each isomer was recorded using a travelling wave ion mobility (TWIM) cell allowing the determination of their respective experimental collision cross section (CCS_exp_). The comparison with the predicted theoretical CCS (CCS_th_) obtained from theoretical calculations propose a regioisomer assignment according to the βCD hydroxyl position (2, 3, or 6) involved in the reaction. These results were validated by extensive NMR structural analyses of pure isomers combined with molecular dynamics simulations. This innovative approach seems to be a promising tool to elucidate complex isomer mixtures such as substituted cyclodextrin derivatives.

## 1. Introduction

Discovered 130 years ago, cyclodextrins (CDs), cyclic oligosaccharides produced by enzymatic degradation of starch, are the most popular macrocyclic molecules. Their inclusion properties associated with their biocompatibility and aqueous solubility have provided them an important impact in many applications fields such as the cosmetic, hygiene, food processing, chemical and pharmaceutical industries or agriculture [1,2]. To improve or to switch their properties, chemical modifications of their different hydroxyl groups were extensively described [3,4,5]. To obtain modified cyclodextrins, two main strategies are available: the first is a regioselective synthesis leading to pure and well-defined cyclodextrin derivatives. Many examples are reported, such as hetero-difunctionalised β-cyclodextrin derivatives [6] or cyclodextrin-based functional glyconanomaterials [7]. Recently, we have proposed an optimised and convergent chemical synthesis of lipophosphoramidyl permethylated βCDs (Lipidyl-βCDs) with various chain lengths [8]. These amphiphilic cyclodextrin derivatives were used successfully to prepare mixed vesicles, but their synthesis requires eight steps and two steps of purification. Probably, the most remarkable example, though it is not so easy to achieve, is the elegant multi-steps synthesis of regioselective multi-hetero-functionalization of CDs described by Sollogoub et al. [9]. This strategy allowed the first synthesis of precisely hetero-functionalised βCDs but required 21 steps with 0.6% overall yield starting from native βCD. This synthesis route is too time-consuming and expensive, and it remains a challenging task that commonly leads to only a low-milligram level of the high-purity compound.

On the other hand, the second synthetic strategy is radically different: few or even one-step and no purification of the different isomers exhibiting various Degrees of Substitution (DS). It seems to be more direct, cheaper and faster. Most of the commercial modified cyclodextrins are obtained with this strategy, such as sulfobutyl-ether βCD (SBEβCD), randomised methylated βCD (RAMEB) or hydroxypropylated βCD (HPβCD). Many modified CDs are also described in the literature, overcoming protection and deprotection steps but leading to complex mixtures of diversely substituted CDs [10,11,12]. For their characterisation, some works focused on the development of liquid chromatography methods to separate mixtures of methylated [13,14] or sulphoalkyl-ether [15,16] derivatives but never access precisely to the structures of the numerous isomers of the same DS. Thus, efficient and rapid characterisation without purification of CD mixtures still remains a challenge since the properties and applications of such compounds are directly linked to the composition of the mixtures obtained.

**Scheme 1:**
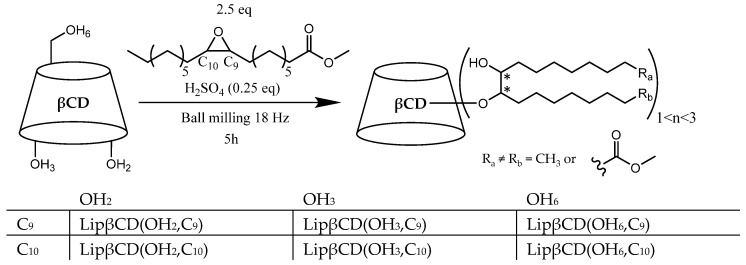
Chemical structure of 12 LipβCD derivatives bearing single lipidyl chain (DS = 1), each isomer named in the table is under racemic form (RR and SS).

**Figure 1:**
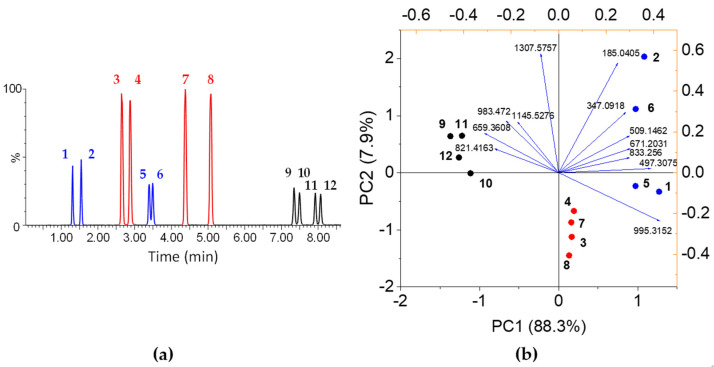
(**a**) Extracted ion chromatogram (EIC) of [LipβCD + Na]^+^ (*m*/*z* 1469.626) obtained fromUHPLC-HRMS separation of the 12 LipβCD isomers of DS = 1. It should be noted the 12 isomers are labelled by their elution order: **1**, **2**, **3**….**12** (**b**) PCA based on MS/MS fragment ions common to all isomers and built with their intensity.

The main goal of this work is to describe and validate an analytical protocol for the characterisation of modified cyclodextrins directly from the crude reaction media. This study is the first attempt to develop a fast and efficient method of complex isomers mixture assignment that will avoid extensive purification and analysis of individual isomers. To set up our analytical workflow, we chose to investigate the DS = 1 isomers mixture obtained from the synthesis of bicatenary biobased lipidyl-cyclodextrins (LipβCD), easily achieved with “one-pot synthesis” from a fatty ester epoxide (Scheme 1) [17]. Following a UHPLC-HRMS (Ultra High-Performance Liquid Chromatography coupled to High-Resolution Mass Spectrometry) analysis, 12 isomers of DS = 1 were observed corresponding to the contribution of the three hydroxyls (OH_2_, OH_3_ and OH_6_) of the βCD which can be involved in the reaction, the two positions of epoxide ring opening (C_9_ and C_10_) and the two configurations (R and S) available for this ring (Scheme 1, Figure 1a). It should be noted that these 12 isomers were labelled on the chromatogram according to their elution order. Thus, this reaction, neither regioselective nor stereoselective, was used as a model.

Our strategy for rapid differentiation of these 12 isomers is based on the respective contribution of MS/MS, ion mobility mass spectrometry (IM-MS) and theoretical calculations. In recent years, IM-MS has proven to be a very promising technique to resolve isobaric ions that are indistinguishable by MS and MS/MS alone [18,19,20]. Indeed, IM-MS can separate isobaric gas-phase ions based on their conformation and shape and can measure the experimental collision cross section (CCS_exp_) of ions to provide information on their intrinsic structures. Furthermore, molecular dynamics techniques were used to explore the conformational space to point out differences between the isomers leading to theoretical collision cross section (CCS_th_). The comparison between CCS_exp_ and CCS_th_ datasets allowed us to assign the different isomers to the chromatographic peaks without purification. Finally, the validation of our attribution required a detailed structural NMR analysis which not only confirmed the results obtained by IM-MS but also provided a better understanding of the structure–activity relationships of the different isomers.

## 2. Results and Discussion

### 2.1. Analytical Workflow for a Rapid Profiling of Cyclodextrins Isomers Mixture

#### 2.1.1. MS/MS Fingerprints Combined with Chemometrics

The detection of the 12 LipβCD isomers of DS = 1 by UHPLC-HRMS separation is depicted in Figure 1a. Firstly, MS/MS acquisitions were performed on their respective [M + Na]^+^ ions (*m*/*z* 1469.6260) using the same collision energy (95 eV). No characteristic fragment of the grafting position was observed in the MS/MS spectra of the 12 isomers. Nevertheless, significant differences in fragment intensities were observed between the isomers (Figures S1 and S2). These intensities were used to build the principal component analysis (PCA), a common multivariate method [21]. Briefly, the main objectives of this approach in analytical chemistry include data reduction, grouping and classification of observations to obtain the modelling of relationships that may exist between variables. This approach allows highlighting, in an unsupervised way, the differences and similarities between the samples, corresponding here to the 12 isomers.

As observed in Figure 1b, this PCA separates the 12 isomers into three groups (black, blue and red), and 88.3% of the variance is explained by this separation on the first component (PC1), confirming the trend of the UHPLC-HRMS chromatogram where three blocks of four peaks seem to be identified according to their polarities and intensities. Regarding the reaction possibilities, these groups could correspond to the three possible grafting positions (OH_2_, OH_3_ or OH_6_) on the βCD. The loadings (i.e., variables = fragments intensities) are also informative, as their placement is responsible for the sample‘s location on the PCA. The loadings corresponding to the fragment ions with the greatest excess mass (due to a large number of H atoms increasing the decimal part of the accurate mass), i.e., the fragments carrying the methyl oleate bicatenary chain, are more expressed in the black group than in the blue one. Conversely, the loadings corresponding to the fragments with the lowest mass excess, i.e., those that have lost the methyl oleate, are more expressed in the blue group than in the black one. According to PC1, the red group is located between the black and blue groups, indicating that carrying the methyl oleate bicatenary chain is not the differentiating criterion for this group. In contrast, the second component (PC2) separates the blue and black groups from the red one. The loadings here indicate that the fragments at *m*/*z* 185.0426 (C_6_H_10_O_5_Na, a sodiated dehydrated glucose adduct) and *m*/*z* 1307.5731 (C_55_H_96_O_33_Na, corresponding to the loss of one dehydrated glucose) seem to explain the difference of this red group compared to the other ones. Indeed, all the variable values in this group are in the middle of those of the black and blue groups, with the exception of these two variables whose values are lower than or equal to those of the other two groups (Figure S3). The involvement of only two variables in this differentiation, according to PC2 and the Pareto scaling, could provide the 7.9% of the variance explained by this component.

#### 2.1.2. Determination of Experimental CCS by IM-MS

To further characterise the 12 LipβCD isomers, TWIMS-type ion mobility was used on a hybrid quadrupole/time-of-flight mass spectrometer (Q-TOF) [22]. This separation technique is located behind the ionisation source, the quadrupole and before the time-of-flight mass analyser, enabling it to be coupled with UHPLC, MS/MS and HRMS. This technique allows the separation of ions by *m*/*z*, charge, shape and size [23]. Thanks to these last two parameters, IM-MS is widely used for the separation of isomers [24,25,26]. Its application in the field of carbohydrate research is steadily increasing for determining the connectivity and the configuration of saccharides [26,27,28]. However, for cyclodextrins, IM-MS is mainly described for the analysis of inclusion complexes and less often for their own characterisation [29,30,31,32]. As mentioned before, another fundamental aspect of ion mobility is that it gives access to the collision cross section (CCS) value, which, when compared to theoretical CCS values (CCS_th_) obtained via molecular modelling techniques, allows for the validation of hypothetical structures. The experimental CCS (CCS_exp_) values are obtained by recording the arrival time (commonly called drift time) distribution (ATD) of each ion that traverses the mobility cell. The arrival time data need to be calibrated first in order to access the CCS_exp_ with an accuracy of ± 2%. Moreover, it should be noted that the solvating effect could be important for the determination of CCS_exp,_ as already reported for macromolecules like proteins [33,34]. Consequently, we investigated whether or not chromatographic elution conditions could influence the ATD in terms of shape, full width at half maximum (FWHM) and area. These experiences allowed us to highlight that no significant differences in ATD were observed according to the composition of the chromatographic mobile phase and so did not affect the CCS_exp_ measurements (Figure S4).

**Figure 2:**
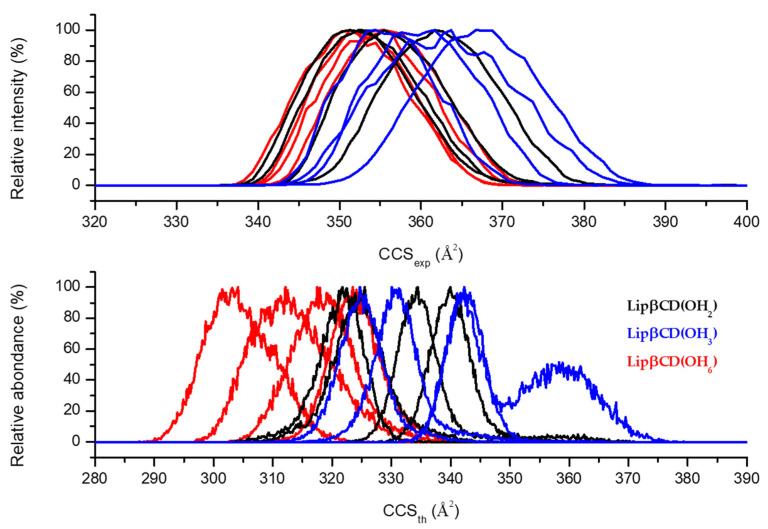
(**Top**): Experimental CCS (CCS_exp_) values distribution obtained from IM-MS experiments. (**Bottom**): Distribution of theoretical CCS (CCS_th_) values computed on an ensemble of minimal structures obtained from metadynamics simulations for the 12 isomers of LipβCD.

In our case, a slight variation of the ATD of DS = 1 isomers was observed; this difference is also the same in terms of CCS_exp_ values (Figure 2, top panel). Thanks to the annotations made by MS/MS (black, blue and red groups of four isomers each), the differences in IM-MS between these groups are relevant. On the one hand, the CCS_exp_ of the blue group is higher and more dispersed. On the other hand, the CCS_exp_ of the red group is smaller and less dispersed. Due to the comparison of a high number of isomers, the prior formation of the groups is, therefore, a prerequisite that allows a more thorough analysis of ion mobility data.

#### 2.1.3. Molecular Metadynamics and CCS_exp_ Assignment of Isomers

To complement the experimental results and to help assignation, molecular dynamics (MD) studies have been undertaken on the 12 isomers in order to gain knowledge on their preferential conformation and to evaluate their theoretical collision cross-section CCS_th_ using the Impact software (for more information on how the CCS_th_ are computed, see reference [35] and references within). Without any knowledge of a stable or most probable conformation adopted by each isomer of LipβCD, we have decided to carry out a conformational search using metadynamics simulations in order to generate large representative ensembles of structures. Furthermore, it has been established that the radius of gyration can be used to evaluate the compactness of a structure [36] and thus can be used to measure differences in CCS_th_. Hence, to explore a vast range of CCS_th_ for any given LipβCD, biased molecular dynamics simulations (i.e., metadynamics simulations) using the gyration tensor as a collective coordinate have been performed.

Once the free energy profile had been reconstructed, we selected structures located within 0.1 Å of the global minimum, which resulted in ensembles of roughly 25,000 conformers for each of the 12 isomers. The results presented in the remainder of this study were obtained via analyses of these ensembles. CCS_th_ values were evaluated using the Trajectory Method (TJM) and the Projection Approximation (PA). The two methods lead to the same distribution of structures but differ in their absolute CCS values (inherent to the methods); see histograms in the supplementary material (Figure S5). In the remainder of the text, only TJM values will be presented, and histograms obtained for the 12 isomers are represented in Figure 2, bottom panel. A first remark is that although (by construction) all the conformers of a given ensemble present a radius of gyration located within a very narrow range (0.2 Å), their CCS values are located in a much wider range. A second remark is that within any given βCD OH substitution position (2, 3 or 6), it is impossible to establish a ranking or draw any conclusion between the four configurations (see Scheme 1). According to the histograms represented in Figure 2 bottom panel, it is obvious that the isomers derived from grafting in position OH_6_ of the lipidyl moiety present the smallest CCS_th_ values, hence are more compact than the eight remaining isomers. The discrimination between isomers grafted on position OH_2_ and OH_3_ is not as obvious, but it seems that OH_3_ isomers present the larger CCS_th_ values of the series. Hence, the following tentative ranking can be established regarding the CCS_th_ values: OH_6_ < OH_2_ < OH_3_. This classification will be used to assign the peaks obtained by IM-MS to the 12 isomers following different hypotheses. It has to be noted that the CCS_th_ and CCS_exp_ values are not directly comparable, as the actual experimental structures are unknown; hence, their coordinates are not available for the CCS_th_ calculation. While the absolute CCS_th_ values are not meaningful, the relative CCS_th_ values are.

Considering the three grafting positions of methyl oleate on βCD (OH_6_, OH_3_ and OH_2_), only six assignment hypotheses are possible (Table 1). The comparison of CCS_exp_ rankings with average $\bar{{\mathrm{CCS}}_{\mathrm{th}}}$ rankings (from largest to smallest) were performed for each hypothesis (for more detailed information, see SI Table S2).

It should be noted that the regioisomer assignment proposed by molecular metadynamics calculations is in agreement with the measurements obtained by IM-MS and with the observations made by UHPLC-HRMS and MS/MS using chemometrics. On the one hand, the βCDs OH_6_ is the most reactive position (57% final yield), and the assigned peaks with higher intensities correspond to this grafting position. On the other hand, MS/MS-based PCA showed a lower loss of methyl oleate upon fragmentation of the assigned OH_2_ of the black group, which is consistent with the orientation of these alcohols towards the interior of the βCD cavity. This approach, using a UHPLC-IM-MS/MS analytical workflow, made it possible—in a single analysis—to assign the regioisomers of methyl oleate grafted βCD (DS = 1) without any purification step. In addition, the contribution of metadynamics allowed for obtaining large ensembles of structures, giving access to a distribution of CCS_th_ and confirming the experimental results.

Moreover, this amount of data may also be interesting for the constitution of a conformational database of CD derivatives. This methodology seems promising because it is fast, allows the discrimination of regioisomers and provides intrinsic data (CCS_th_) and a large amount of theoretical information. Nevertheless, the experimental data (CCS_exp_) are quite close and need a validation step to verify this methodology. For this purpose, following an individual purification of the 12 isomers, our regioisomers assignment will be validated by molecular modelling and NMR studies. Comparing CCS_exp_ and $\bar{{\mathrm{CCS}}_{\mathrm{th}}}$ values have led to an attribution of the grafting position for the 12 chromatographic peaks without discrepancies between the two methods (experimental measurements vs. theoretical calculations). However, to definitely validate this attribution, on the one hand, and to be able to differentiate one isomer among the twelve, on the other hand, further analyses to obtain 3D information on the structures have to be performed.

### 2.2. Validation of the Method

#### 2.2.1. Molecular Metadynamics Studies

**Figure 3:**
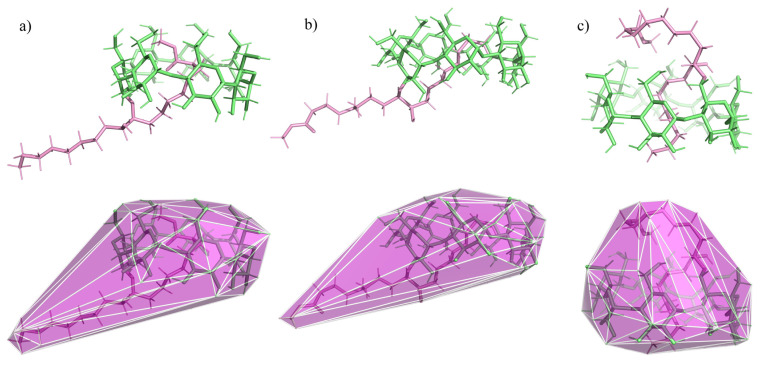
Representative structures of LipβCD substituted in (**a**) position 2, LipβCD(OH_2_), (**b**) position 3 LipβCD(OH_3_) and (**c**) position 6, LipβCD(OH_6_).

In order to gain a better understanding of the ranking established in the previous section and to validate it, further analyses to evaluate the shape and compactness of the different isomers have been undertaken such as a calculation of the geometrical surface and volume of the substituted-CD, on the one hand, and anisotropy of the radius of gyration, on the other hand. Moreover, the conformational space corresponding to the free energy well bottom was analysed, and representative structures of each isomer were obtained by clustering the sampled conformations into ten representative classes. The ~25,000 structures corresponding to the free energy well bottom, although featuring the same radius of gyration plus or minus 0.1 Å, adopt multiple conformations. Clustering analyses showed that the most representative structure for each of the twelve isomers is an auto-inclusion conformer: either the alkyl arm or the ester arm is present in the cavity, which is consistent with experimental results presented in the next section, see Figure 3 for a general representation of each substitution. As, by construction, it is easier for the two lipidyl chains grafted in position 6, LipβCD(OH_6_), to interact with the cavity, the histograms relative to these conformers in Figure 2 (bottom panel) are moderately stretched, corresponding to the different interactions possible for the second arm around the CD scaffold. Histograms for LipβCDs grafted in position 2 or 3, LipβCD(OH_2_) and LipβCD(OH_3_), are narrower (except for one) but are located at higher CCS values. The most representative structures for a lower rim substitution show that one lipidyl chain is present in the cavity, while the second one is away, i.e., does not interact with it. As this latter does not fold around the CD scaffold, not only is the CCS higher, but also the possible number of conformations is lower compared to grafting in position 6.

Geometrical surfaces and volumes for the twelve different isomers can be found in Table 2. Again, without ambiguity, CDs grafted in position 6 present the smallest values, thus proving that their structures are the most compact of the whole series of isomers. The difference in values between LipβCD(OH_2_) and LipβCD(OH_3_) is slim, but structures corresponding to grafting in position 3 seem to be the largest. These trends totally agree with the ranking established with the CCS values. Table 2 also shows the average anisotropy or asphericity of the radius of gyration for the twelve isomer families, which lies between 0 (for spheres) and 1 (for rods). It has to be reminded that for each isomer, the selected structures show a nearly constant radius of gyration (variation of ± 0.1 Å). Structures corresponding to a grafting in position 6 are the more spherical, whereas structures corresponding to a grafting in position 3 are the more elliptical, validating again the previously established ranking.

This ranking is consistent with global cyclodextrin chemistry and sterical hindrance. Indeed, grafting in position 6 results in more flexible structures: compared to positions 2 and 3, position 6 offers an additional -CH_2_- function resulting in more freedom and less hindrance for the grafted moiety, hence less deformation of the CD cavity to accommodate for the inclusion of one of the lipidyl chain through the upper and narrow rim, the second arm then being able to interact with the external scaffold of the CD. The LipβCD(OH_6_) structures are globally spherical and compact. Grafting in positions 2 and 3 is less readily compatible with self-inclusion (occurring at the lower and larger rim), and the CD cavity needs to adapt to welcome the lipidyl chain, while the second arm is extended and unable to interact with the CD scaffold. The LipβCD(OH_2,3_) geometries are globally more stretched, resulting in more elliptical and deformed structures. The difference between positions 2 and 3 lies in the orientation of the -OH function: OH_2_ points “inward”, whereas OH_3_ points “outward”, leading to a lesser sterical hindrance upon self-inclusion of the lipidyl chain for the position 2, and a more important deformation of the cavity for CDs substituted at position 3.

A molecular metadynamics study of the 12 isomers of LipβCD concluded that for each of them, the most probable/stable structure in water corresponds to a structure where one lipidyl chain is inserted into the cavity. Depending on the grafting position of the lipidyl onto the CD, resulting structures show different compactness leading to different CCS_th_. Histograms obtained through calculations are quite broad (Figure 2, bottom panel), discarding the presence of a unique conformer. Additionally, experimental arrival time distributions are also broad, indicating the presence of multiple structures with different mobilities and cross sections. In this context, calculations are in agreement with experimental results, and the combination of these two methods allowed for establishing without ambiguity the following rankings for CCS_th_: LipβCD(OH_6_) < LipβCD(OH_2_) < LipβCD(OH_3_). As is, the molecular modelling study presented in this work is unable to draw significant trends regarding the substitution position (C_9_ and C_10_) and the chirality (RR or SS) of the connecting carbon atoms. Additional analyses and classical MD simulations (i.e., without any bias) to account for the inclusion of the lipidyl chains accompanied by quantum ab initio calculations (geometry optimizations, energies, NMR chemical shifts, etc.) are mandatory to help the assignation of the different peaks obtained through LC-MS experiments. These studies will be the scope of a forthcoming article.

#### 2.2.2. NMR Studies

**Figure 4:**
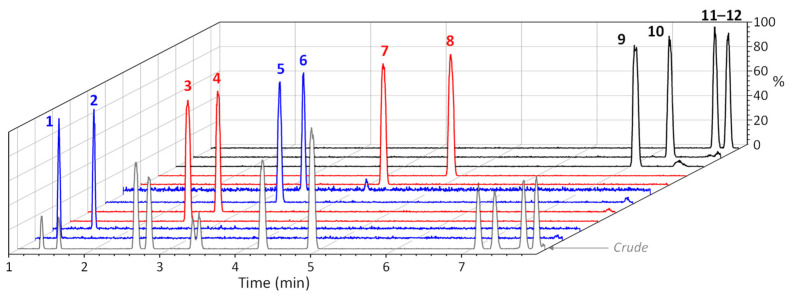
UHPLC-HRMS chromatograms (EIC *m*/*z* 1469.6260) of the isomers mixture and of the 11 purified fractions obtained by preparative chromatography. It should be noted the 12 isomers are labelled by their elution order: **1**, **2**, **3**….**12**.

A preliminary step to NMR characterisation was the purification of the DS = 1 isomers mixture, which was led with an auto-purification system after the transposition of the UHPLC conditions (Figure S6). Due to the larger dimensions of the column—and a loss of chromatographic resolution—10 out of 12 pure isomers were obtained. The collected fractions were analysed by UHPLC-HRMS to highlight the purity of each fraction. Isomers corresponding to peaks **1** to **10** were pure, whereas those corresponding to peaks **11**–**12** were obtained in a mixture (Figure 4).

**Figure 5:**
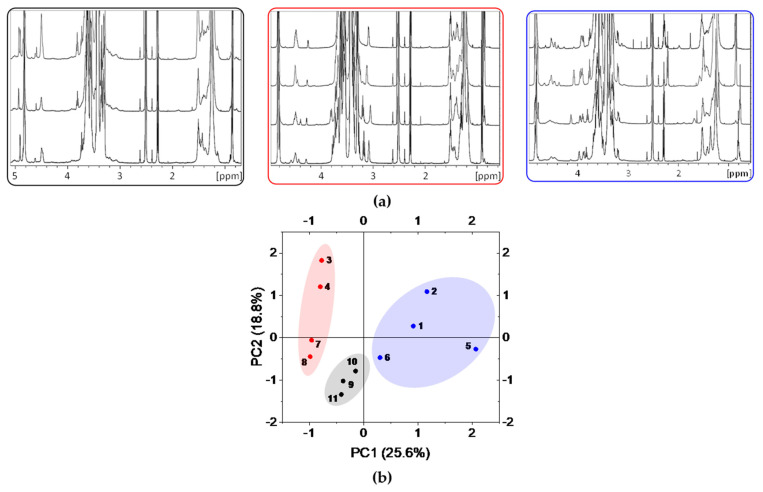
(**a**) ^1^H NMR spectra (600 MHz, DMSO/D_2_O 98/2 *v*/*v*, 298 K) of the purified isomers: blue group (from bottom to top: **1**, **2**, **5**, **6**), red group (**3**, **4**, **7**, **8**) and black group (**9**, **10**, **11**−**12**). (**b**): PCA based on chemical shifts of ^1^H spectra.

**Figure 6:**
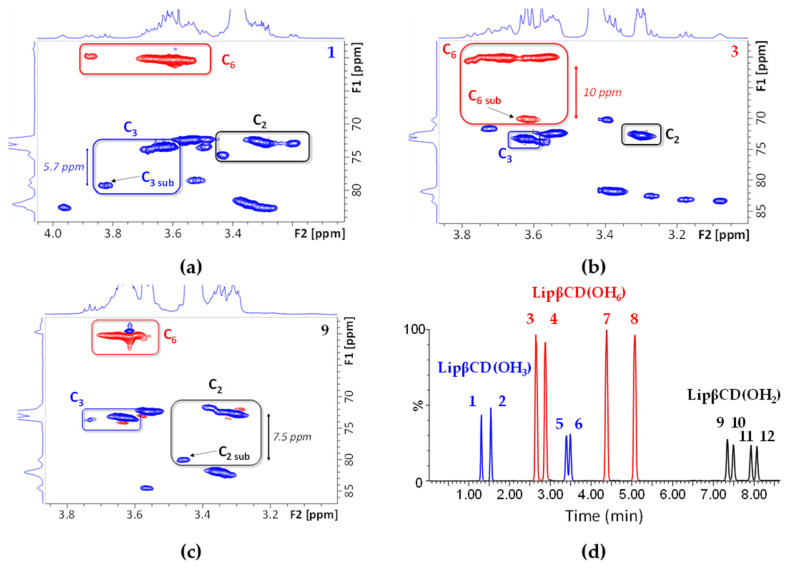
Partial contour plot of ^1^H-^13^C HSQC experiments (600 MHz, DMSO/D_2_O 98/2, *v*/*v*, 298 K) of isomers **1** (**a**), **3** (**b**) and **9** (**c**). (**d**) EIC of LipβCD marked with the regioisomers assignment based on HSQC experiments.

A complete NMR characterisation was carried out in DMSO/D_2_O (98/2, *v*/*v*) on the 12 pure isomers involving NMR experiments such as COSY, TOCSY, HSQC or HMBC. For the sake of clarity, only the three most relevant points are described here. The first point is the comparison of the ^1^H NMR experiments performed on each isomer in the same experimental conditions, as displayed in Figure 5. As expected for modified cyclodextrin derivatives, the obtained ^1^H NMR spectra are very complex, and too many overlapping signals prevent any direct attribution. Nevertheless, some similarities in terms of chemical shifts can be observed, leading to the spectra’s classification into three groups named blue, red and black (Figure 5a and Figure S7). Comparison of spectra of three compounds (**1**, **3**, **9**) from each different group clearly shows differences in terms of chemical shifts (see Figure S8). Another approach to explore the NMR chemical shift data was PCA methods using the variation of chemical shifts. The three same groups are observed in agreement with MS/MS study (Figure 5b). To go deeper and to try to assign each isomer, three compounds (one from each group) were selected: the compounds named **1**, **3** and **9**. The complete assignment of all carbon signals is achieved by coupling the COSY, TOCSY and HSQC experiments. HSQC experiment displayed in Figure 6 seems to be the most relevant NMR experiment to determine the grafting position on OH_3_, OH_6_ or OH_2_ of βCD for each isomer. The specific chemical shift of single carbon of CD is observed for each compound: C_3_ for isomer **1** of the blue group, C_6_ for isomer **3** of the red group and, finally, C_2_ for isomer **9** of the black group. The important variations of the ^13^C chemical shift observed in the three cases (5.7, 10 and 7.5 ppm, respectively) are in agreement with a substitution on OH_3_, OH_6_ and OH_2_ accordingly [6,8]. The same phenomenon is observed for the other compounds of the same group (**2**, **5** and **6** for the blue group, **4**, **7** and **8** for red group and **10**–**12** for the black group) (See Figures S9–S11).

These results are the same as those obtained by the IM-MS hypothesis combined with molecular modelling. This latter approach performed on a mixture without purification was therefore validated by an NMR study carried out after purification. In addition, access to pure isomers allows the assigning of the grafted carbon (C_9_ and C_10_) on the lipidyl chain by NMR study. For that, two isomers of the same group were selected, the compounds **3** and **4** of the red group corresponding to LipβCD(OH_6_).

**Figure 7:**
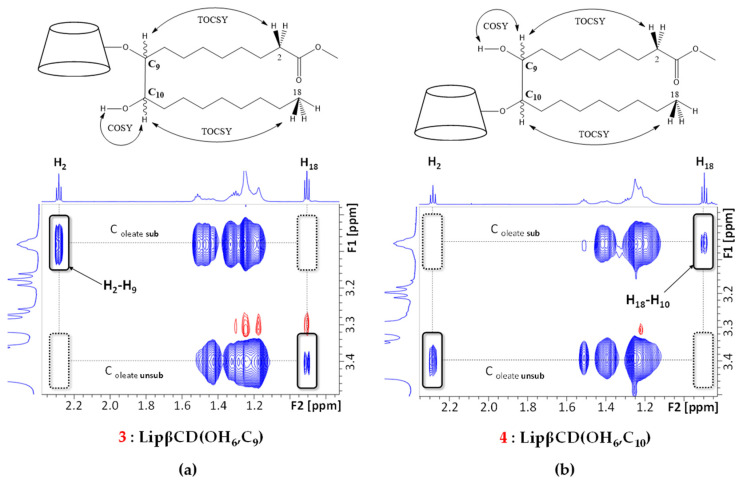
Partial contour plots of 2D TOCSY experiments (600 MHz, DMSO/D_2_O 98/2, *v*/*v*, 298 K, t_m_ = 160 ms) of compounds **3** (**a**) and **4** (**b**). The strategy to determine the grafted position of the methyl oleate carbon is shown at the top of the contour plots.

**Figure 8:**
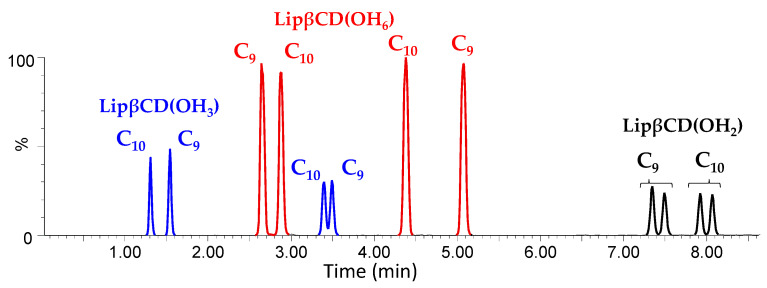
Final assignment of the 12 chromatographic peaks obtained by UHPLC-HRMS, described by the grafted position on βCD (OH_2_, OH_3_ and OH_6_) and on methyl oleate (C_9_, C_10_).

TOCSY NMR experiments were performed on isomers **3** and **4** (Figure 7). Note two interesting cross peaks, obtained with a long mixing time (t_m_) as observed in Figure S12 and involving the H_2_ proton and the methyl 18, located on each lipidyl chain. For both isomers, we can observe a correlation between H_2_ and proton H_9_ and between methyl 18 and proton H_10_. The discriminating element is the following: for isomer **3**, the free OH, obtained after the epoxide ring reaction and observed in pure DMSO (data not shown), is correlated with H_10,_ and in contrast, for isomer **4** with H_9_. It is possible to conclude that isomer **3** is grafted by βCD in position 9 and isomer **4** in 10, indicating that **3** is LipβCD(OH_6_, C_9_) and **4** is LipβCD(OH_6_, C_10_). The same strategy was applied to all other isomers to obtain the following final assignment, as shown in Figure 8.

**Figure 9:**
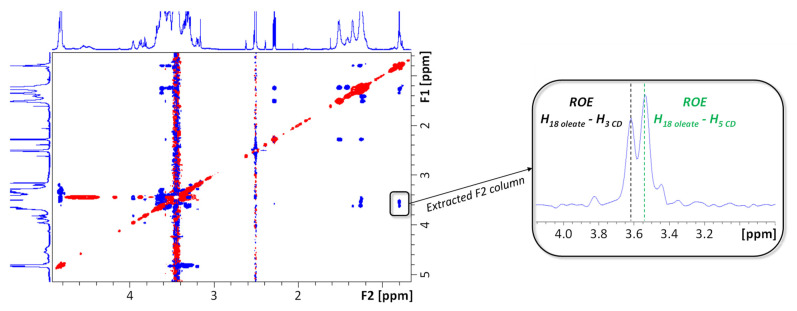
ROESY NMR experiment (600 MHz, DMSO/D_2_O 98/2, *v*/*v*, 298 K, t_m_ = 800 ms) of isomer **1**. The extracted columns containing dipolar correlations with H_18oleate_ (methyl, δ = 0.8 ppm) are inserted and show cross-peak correlations with H_3CD_ and H_5CD_, highlighted by black and green dotted lines, respectively.

The study of ROESY experiments carried out on the 12 isomers in a DMSO/D_2_O (98/2, *v*/*v*) mixture shows numerous cross-peak correlations between the lipid parts and the cyclodextrin cavity, although DMSO is well known to have a strong affinity for the cavity of CDs and to inhibit complex formation (Figure S13). It should be noted that in all cases, interactions between any proton of the bicatenary lipidyl chain and inner protons of the cyclodextrin are observed. As most of the signals are overlapping, we focus on the interactions between methyl 18 of the lipidyl chain and protons located in the cavity as observed in Figure 9 for isomer **1**, named LipβCD(OH_3_, C_10_). The assignment is achieved through HSQC experiences, and Figure 9 evidences spatial proximity between methyl 18 and H_3_ and H_5_ protons in agreement with the inclusion of this lipidyl chain inside the cavity.

The same treatment is applied to the other isomers, and the obtained results are summarised in Figure S14. Once again, isomers belonging to one group provide similar results demonstrating that the grafting position on the CDs seems to be a driving force of the compound’s three-dimensional structure. In the case of the LipβCD(OH_2_) group, methyl 18 exhibits weak interactions with CDs cavity in accordance with the corresponding lipidyl chain located outside the cavity (Figure 9). For LipβCD(OH_3_) and LipβCD(OH_6_) groups, two out of every four terminal methyl 18 are located inside the CD cavity (blue and red curves) but close to H_5_ and H_3,_ respectively (Figure S14b,c), suggesting that the resulting lipidyl chain is in the cavity in both cases. The presence of self-inclusion (intramolecular) rather than inclusion complex (intermolecular) phenomena is supported by the DOSY NMR experiments performed in DMSO at different concentrations. The diffusion coefficient *D* values do not vary significantly (from 1.18 to 1.24 × 10^−10^ m^2^·s^−1^) when the concentration of the sample in DMSO is increased by a factor of 10 (Figure S15).

## 3. Materials and Methods

### 3.1. Chemicals

The Lipidyl cyclodextrins LipβCD were obtained by an in-home synthesis described in our previous work [17]. β-cyclodextrin (βCD, Cavamax W7 Pharma) was supplied by Wacker Chemie AG (Munich, Germany). Deuterated water and DMSO were purchased from Sigma-Aldrich. The water used was MilliQ grade. All solvents used were analytical grade.

### 3.2. UHPLC, HRMS, MS/MS and IM-MS

Analyses were performed with a UPLC H-Class chromatographic system in line with a Synapt-G2-Si hybrid Q-TOF mass spectrometer (Waters, Manchester, UK) equipped with an ESI source ionisation and a travelling wave ion mobility cell (TWIM).

UHPLC conditions: Samples were prepared in MeOH at a concentration of 1 mg·mL^−1^ (≈ 50 μM), and 1 μL was injected. For UHPLC separations, an ACQUITY UPLC BEH C8 (100 mm, 2.1 mm, 1.8 μm) column maintained at 40 °C was used. The elution was performed using a 0.4 mL·min^−1^ mobile phase gradient of water (A) and methanol (B), programmed as follows (A:B): 60:40 (t = 0 min), 20:80 (t = 10 min), 20:80 (t = 11 min), 0:100 (t = 12 min), 0:100 (t = 15 min), 60:40 (t = 16 min), 60:40 (t = 20 min).

The ESI source was operated in the positive ionisation mode using a capillary voltage of +3 kV and the following conditions: cone voltage, 120 V; source offset, 20 V; source temperature, 120 °C; desolvation gas temperature, 450 °C; desolvation gas flow, 800 L·h^−1^; and cone gas flow, 50 L·h^−1^. Nitrogen (>99.5%) was employed as the desolvation gas. Mass calibration was carried out using a sodium formate solution (10 mM NaOH in isopropanol/water/formic acid 49.9:49.9:0.2, *v*/*v*/*v*), and the lock mass correction was applied for accurate mass measurements using a Leu-enkephalin solution (*m*/*z* 556.2771). The scan range was *m*/*z* 50–2000 at 0.20 s·scan^−1^. The TOF was operated in the resolution mode, providing an average resolving power of 25,000 (FWHM). For MS/MS, spectra were recorded using the trap cell, with collision energy set to 95 eV under Argon (99.9999%) as the collision gas. All spectra were recorded in the continuum mode.

Ion mobility: ion mobility separation has been carried out with a TWIM cell operating under nitrogen (>99.5%) with wave height (WH), wave velocity (WV), and gas flow values at 40 V, 550 m·s^−1^ and 90 mL·min^−1^, respectively. The mass of DS = 1 ([M + Na]^+^ *m*/*z* 1469.6260) was previously selected by the quadrupole to reduce noise. The TWIM cell needs to be calibrated to obtain collision cross section (CCS) values from drift time [23]; calibration was performed using a MajorMix solution (Waters). Data acquisition was performed with the MassLynx software (V4.2, Waters). The arrival time (AT) of each ion of interest (calibration ions and CDs ions) was extracted with the Driftscope software (V2.9, Waters), and apex was determined by a gaussian fit performed with the Origin software (v2019, OriginLab Corporation, Northampton, MA, USA).

Direct introduction conditions: the purified isomers (**1**, **4** and **10**) and natural CDs (αCD, βCD and γCD) were solubilised at 5 μM in different solvents: H_2_O 100%, H_2_O/MeOH 50/50 *v*/*v* and MeOH 100%. Each sample (3 per CD) was analysed by flow injection analysis (FIA) using isocratic H_2_O 100%, H_2_O/MeOH 50/50 *v*/*v* or MeOH 100% as the mobile phase with a flow rate of 0.4 mL·min^−1^. All mass spectrometer and ion mobility parameters were the same as for UHPLC analyses.

To control the calibration efficiency, natural CDs were analysed, and the Δ CCS obtained ([M + Na]^+^) were below 2% in comparison with CCS obtained with DTIMS in the literature (Table S1) [37,38].

### 3.3. Computational Details

Initial geometries for the twelve isomers of lipidyl-βCDs studied in this work were built using the LEaP program from the AmberTools16 distribution [39]. The fragments needed to build the native βCDs were taken from the R.E.DD.B. database [40] under project F-85, and the fragments needed to construct the lipidyl arm were parameterised and defined according to the general strategy we developed previously for substituted cyclodextrins [41]. All molecular dynamics simulations were carried out using the SANDER module from the AmberTools16 program suite [39]. The twelve LipβCD isomers were solvated in a truncated octahedral box with a buffer distance of 10.0 Å, which represents 1900 to 2500 molecules of water, depending on the shape of each isomer. The q4md-CD force field parameters [41] were used to model the βCD scaffold, and the AMBER99SB force field [42] was used for the lipidyl substituent. The parameters used for water were taken from the TIP3P model [43]. After minimization, the systems were brought to target temperature by ramping up the temperature over periods of 25 ps followed by a run of 200 ps to relax and equilibrate the system and short classical MD simulations of 5 ns were performed using the NPT ensemble at a pressure of 1 atm and a temperature of 300 K. Finally, metadynamics simulations [44] of 300 ns were then conducted; all biasing to molecular dynamics and definition of collective coordinates were carried out using the open-source, community-developed PLUMED library [45], version 2.4.2. [46]. Metadynamics placed potentials of the initial height of 0.3 kcal·mol^−1^ and width of 0.1 Å for the radius of gyration every 100 steps applying a bias factor of 20. Throughout the simulations, the weak coupling algorithm [47] was used to regulate the temperature and pressure to 398 K and 1 atm, respectively. The temperature was maintained close to the intended value by weak coupling to an external temperature bath with a relaxation time of 2 ps and the pressure to an external pressure bath of 1 atm with a coupling constant of 2 ps. The SHAKE algorithm [48] was used to constrain C-H bonds, and a time step of 2 fs was used to integrate the equations of motion. Periodic boundary conditions were imposed during simulation. The distance cutoff of 9.0 Å was applied to non-bonded interactions, and the PME method [49] was used to compute long-range interactions. Configurations of the systems were stored at intervals of 1 ps. Analyses and clustering of the trajectories were performed using the Cpptraj module [50] available in the AmberTools distribution. The clustering analysis was powered by the hierarchical agglomerative approach using LipβCD RMS distances. The Qhull program [51] was used to evaluate the geometrical surface and the volume of the LipβCD structures, and CCS_th_ values were calculated with the Impact software using the trajectory method and the projection approximation [35]. These CCS_th_ values have been calculated on structures extracted from MD simulations in explicit water, hence presenting a solvated conformation. It has been shown in the literature that for comparisons with IM-MS work performed in vacuum, this strategy is valid because, during the evaporation phase, CDs should retain their native solution structure [52,53]. Furthermore, this assumption has been experimentally verified in this work (Figure S4). However, direct comparisons between theoretical calculations of CCS_th_ and values obtained through IM-MS experiments are not advisable, as the theoretical structures lack the presence of the Na^+^ ion.

### 3.4. Purification Method

Isomers purification was performed with auto-purification system with a 1525 binary pump coupled with a single quadrupole mass spectrometer SQD2 (Waters-Micromass, Manchester, UK) equipped with an electrospray ion source (ESI-MS). The separation was performed at room temperature on a Fuji SPS-300-5-C8T (250 mm, 25 mm, 5 μm) column using a sample injection volume of 600 μL (solution of isomers mixture at 50 g·L^−1^ in DMSO). The following mobile phase gradient at a flow rate of 23 mL·min^−1^ was used: (A) H_2_O, (B) MeOH; (A:B): 70:30 (t = 0 min), 30:70 (t = 20 min), 30:70 (t = 30 min), 0:100 (t = 30.1 min), 0:100 (t = 40 min), 70:30 (t = 40.10 min), 70:30 (t = 50 min). An in-line splitter was set at 1/1000 to reduce the flow toward the ESI-MS source. The ESI source was operated in the positive ionisation mode using a capillary voltage of +3 kV and the following conditions: cone voltage, 60 V; source temperature, 150 °C; desolvation gas temperature, 350 °C; desolvation gas flow, 650 L·h^−1^; and cone gas flow, 20 L·h^−1^. Nitrogen (>99.5%) was employed as the desolvation gas. Both [M + Na]^+^ (*m*/*z* 1470) and [M + 2Na]^2+^ (*m*/*z* 746) were used to detect and auto-collect DS = 1 isomers. Data acquisition and processing were performed with the MassLynx V4.1 software.

### 3.5. Nuclear Magnetic Resonance (NMR) Analyses

NMR experiments were performed on an AVANCE III 600 MHz spectrometer (Bruker, Wissembourg, France) equipped with a Z-gradient unit (for pulsed-field gradient spectroscopy) and a triple resonance probe (TXI, 5-mm tube). Spectra were acquired at 298 K with close temperature control. DMSO-d6 was used as the solvent, with D_2_O 2% to exchange free H of CDs free alcohol groups. The system’s pulsecal automation program was used to optimise the duration of the 90° pulse. Residual ^1^H signal of deuterated solvent was used as the reference for calibration. One-dimensional NMR spectra were recorded at a resolution of 0.12 Hz (64 K data points). ^1^H spectra were obtained with the Bruker sequence zg30. TOCSY experiments were performed using the Bruker sequence mlevph, mixing time was set as 160 ms; F1 and F2 resolutions are 37.5 Hz and 4.7 Hz, respectively. COSY, relayed COSY and HSQC experiments were carried out using standard sequences of Bruker library. 2D ROESY ^1^H NMR experiments were carried out using the phase-sensitive roesyph.2 sequence; the mixing time was set to 800 ms, with resolutions of 2.3 Hz and 18.7 Hz for F2 and F1, respectively. The TopSpin software (Bruker) v3.8 was used for acquisition, and v4.0.7 was used for data treatment.

Some NMR experiments were realised on an AVANCE NEO 900 MHz to gain in resolution and sensitivity. DMSO-d_6_ 100% was used as solvent, allowing the observation of alcohol with good resolution. COSY and HSQC experiments were performed using the Bruker sequence cosygpqf and hsqcedetgpsisp (v2.4), respectively.

### 3.6. PCA Analyses

MS/MS data treatment: Followed shared ions fragment obtained by MS/MS ([M + Na]^+^, *m*/*z* 1469.6260) for the 12 isomers were used: 185.0405, 347.0918, 497.3075, 509.1462, 659.3608, 671.2031, 821.4163, 833.256, 983.472, 995.3152, 1145.5276, 1307.5757. Their intensity (I) was pretreated as follows: $I\;\to \;log\left(\frac{I}{I_{\;parent}}\right)$. PCA was realised with Pareto scaling to keep small variance’s variations between variables.

NMR data: ^1^H NMR spectra (600 MHz, 128 scans, D1 = 18 s, 298 K) were recorded with zg pulseprog (600 MHz, Bruker) with larger spectral window (SW = 14 ppm, O1P = 4.7 ppm) to optimise baseline. NMRProcFlow tool (v1.4.16) [54] was used to obtain homogenous baseline correction (global correction, noise windows: 11.5–9.0 ppm) on previously phased spectra. From these spectra, NMRprocflow automatically generates and integrates buckets of 0.04 ppm at the following defined windows: 4.95–4.72 (H_1CD_), 4.15–3.48 (H_3_, H_5_, H_6CD_; H_9–10Oleate_), 3.39–3.0 (H_2_, H_4 CD_; H_9–10Oleate_), 2.34–2.14 (H_2Oleate_), 1.6–1.05 (H of CH_2Oleate_), 0.95–0.72 (H_18Oleate_). For each spectrum, the integration values of buckets were normalised to the sum of the integrations to constitute the variables. PCA was performed with Unit-Variance (UV, standard distribution) scaling to keep same contribution of each window, i.e., to avoid strong contribution of methyl singlet.

PCA was realised with the Origin software (v2019, OriginLab Corporation, Northampton, MA, USA).

## 4. Conclusions

A complex isomers mixture of Lipidyl-βCDs (LipβCD) obtained with DS = 1 from a one-step synthesis pathway has been fully characterised without previous purification. An assignment of the regioisomers, according to the grafting positions of the lipidyl chain on the βCD hydroxyls, was achieved unambiguously based on a combination of IM-MS measurements and a theoretical approach. These results were validated by extensive NMR structural analyses of pure isomers combined with molecular metadynamics simulations. This innovative approach seems to be a promising tool to elucidate complex isomer mixtures such as substituted cyclodextrin derivatives. Moreover, conformational searches performed through metadynamics provided a large amount of structures, which, when confronted with NMR studies, will lead to the elucidation of many structure–function relationships such as solubility, inclusion or self-assembly properties. These studies will be the scope of a forthcoming article.

## Figures and Tables

**Table 1:** Score (%) obtained with the comparison of CCS_exp_ and $\bar{{\mathrm{CCS}}_{\mathrm{th}}}$ for the assignment hypotheses of the grafted position on βCD. The blue, red and black colors correspond to the group of regioisomers defined by MS/MS experiments.

	Hypotheses					
Group	H1	H2	H3	H4	H5	H6
Blue	OH_3_	OH_3_	OH_2_	OH_2_	OH_6_	OH_6_
Red	OH_6_	OH_2_	OH_3_	OH_6_	OH_3_	OH_2_
Black	OH_2_	OH_6_	OH_6_	OH_3_	OH_2_	OH_3_
Score	82%	69%	40%	47%	20%	15%

**Table 2:** Average surfaces, volumes and average anisotropies of the radius of gyration tensor computed for the 12 families of LipβCD isomers. A value of zero corresponds to a sphere-like structure, and a value of one to a rod-like structure.

	Surface (Å^2^)	Volume (Å^3^)	Asphericity/Anisotropy
LipβCD (OH_2_, C_9_)	700.73 ± 28.10	1428.13 ± 95.33	0.370 ± 0.054
LipβCD (OH_2_, C_10_)	705.27 ± 38.98	1440.23 ± 109.34	0.366 ± 0.054
LipβCD (OH_3_, C_9_)	732.88 ± 42.19	1494.47 ± 136.29	0.411 ± 0.060
LipβCD (OH_3_, C_10_)	714.02 ± 36.32	1452.30 ± 107.88	0.400 ± 0.058
LipβCD (OH_6_, C_9_)	636.91 ± 30.81	1307.65 ± 85.63	0.268 ± 0.038
LipβCD (OH_6_, C_10_)	664.86 ± 33.96	1372.59 ± 97.04	0.283 ± 0.041

## Data Availability

The data presented in this study are available on request from the corresponding authors.

## Supplementary Materials

The following supporting information can be downloaded at: https://www.mdpi.com/article/10.3390/ijms232113352/s1.
