# Supplementary material for: Mass Spectrometry, Ion Mobility Separation and Molecular Modelling: A Powerful Combination for the Structural Characterisation of Substituted Cyclodextrins Mixtures
Source: Int J Mol Sci. 2022 Nov 1;23(21):13352. doi: 10.3390/ijms232113352 (PMC9656358; doi:10.3390/ijms232113352)
Supplement: Supplementary file 1 [file ijms-23-13352-s001.zip › ijms-1973389-supplementary.pdf]

International Journal of
Molecular Sciences

MDPI

Article

Mass Spectrometry, Ion Mobility Separation and Molecular Modelling: A Powerful Combination for the Structural Characterisation of Substituted Cyclodextrins Mixtures

Sébastien Rigaud [1], Abdouramane Dosso [1,†], David Lesur [1], Dominique Cailleu [2], David Mathiron [2], Serge Pilard [2], Christine Cézard [1] and Florence Djedaini-Pilard [1,*]

[1] Laboratoire de Glycochimie des Antimicrobiens et des Agroressources, UMR CNRS 7378, Institut de Chimie de Picardie, FR CNRS 3085, F-80039 Amiens, France; sebastien.rigaud@u-picardie.fr (S.R.); abdouramane.dosso@agroparistech.fr (A.D.); david.lesur@u-picardie.fr (D.L.) ; christine.cezard@u-picardie.fr (C.C.)

[2] Plateforme-Analytique, Institut de Chimie de Picardie, FR CNRS 3085, Université de Picardie Jules Verne, F-80039 Amiens, France; dominique.cailleu@u-picardie.fr (D.C.); david.mathiron@u-picardie.fr (D.M.); serge.pilard@u-picardie.fr (S.P.)

* Correspondence: florence.pilard@u-picardie.fr

† Present address: URB ABI, AgroParisTech, 3 rue des Rouges Terres, F-51110 Pomacle, France.

Supplementary Materials: The following are available online at https://www.mdpi.com/article/10.3390/ijms232113352/s1,

Figure S1. MS/MS spectra of [LipβCD+Na]$^+$ (m/z 1469.63, EC = 95 eV) corresponding to the blue group isomers : **1 (a)**, **2 (b)**, **5 (c)** and **6 (d)**. **Figure S2.** MS/MS spectra of [LipβCD+Na]$^+$ (m/z 1469.63, EC = 95 eV) corresponding to one isomer of each group: blue group **1 (a)**, red group **3 (b)** and black group **9 (c)**. Note that spectra are identical for **(a)** compounds **1**, **2**, **5**, **6**; **(b)** compounds **3**, **4**, **7** and **8**; **(c)** compounds **9**, **10**, **11** and **12**. **Figure S3.** Raw data used to build the PCA, obtained from the intensities of fragment ions observed in MS/MS spectra. **Figure 4.** The descriptive values CCS (black), FWHM (blue) and area (red) of ion mobility distributions obtained with different solvents conditions (in-flow solvent and sample solvation) for native βCD **(a)** and LipβCD(OH_6) isomer **4 (b)**. **(c)** Superimposition of the arrival time distributions obtained by the in-flow injection analysis (FIA) of pure native βCD and LipβCD(OH_6) isomer **4**, diluted in H_2O/MeOH 50/50 *v/v* and push with H_2O (blue), H_2O/MeOH 50/50 *v/v* (red) and MeOH (black) as in-flow solvent. **(d)** Comparison of CCS values obtained by UHPLC-IM-MS of the mixture (black) and by FIA-IM-MS of each pure isomers with H_2O/MeOH 50/50 *v/v* as in-flow solvent (red). **Figure S5.** Distribution of theoretical CCS_{th} values obtained for the minimal structures of the twelve isomers of LipβCD using the Trajectory Method and the Projection Approximation. **Figure S6.** EIC of [LipβCD+Na]$^+$ (m/z 1469) obtained from preparative scale LC-MS. **Figure S7.** ^{1}H NMR spectra (600 MHz, DMSO/D_2O 98/2, *v/v*, 298 K) of isomers **3**, **4**, **7** and **8** (from bottom to top). The expansion of the spectral area (2.9 - 3.9 ppm) highlights the similarity of the spectra in the same group (red group here). The stars indicate the proton of C_{oleate} grafted on βCD. **Figure S8.** ^{1}H NMR spectra (600 MHz, DMSO/D_2O 98/2, *v/v*, 298 K) of isomers **1**, **3** and **9** (from bottom to top). The expansion of the spectral area (2.9 – 4.2 ppm) highlights the spectra differences between the groups. The stars indicate the proton of C_{oleate} grafted on βCD and the colors their corresponding group. **Figure S9.** Partial contour plot of HSQC experiments (600 MHz, DMSO/D_2O 98/2, *v/v*, 298 K) of isomers **3 (a)**, **4 (b)**, **7 (c)** and **8 (d)** of the same LipβCD(OH_6) red group. **Figure S10.** Partial contour plot of HSQC experiments (600 MHz, DMSO/D_2O 98/2, *v/v*, 298 K) of isomers **9 (a)**, **10 (b)** and **11-12 (c)** of the same LipβCD(OH_2) black group. **(d)** Superimposition of partial contour plot of HSQC and HMBC experiments of the **11-12** isomer mixture (arbitrarily noted *a* and *b*). **Figure S11.** Partial contour plot of HSQC experiments (600 MHz, DMSO/D_2O 98/2, *v/v*, 298 K) of isomers **1 (a)**, **2 (b)**, **5 (c)** and **6 (d)** of the same LipβCD(OH_3) blue group. **Figure S12.** 2D TOCSY experiments (600 MHz, DMSO/D_2O 98/2, *v/v*, 298 K) of isomer **3** performed with a mixing time of 100 ms **(a)** or 160 ms **(b)**. The presence or absence of long distance correlation peaks is highlighted by solid or dash rectangles. **Figure S13.** 2D ROESY experiments (600 MHz, DMSO/D_2O, *v/v*, 298 K 98/2, t_m = 800 ms) of isomers **1- 12**. **Figure S14.** Superimposition of extracted columns from ROESY NMR experiments (600 MHz, DMSO/D_2O 98/2, v/v, 298 K, tm = 800 ms) containing dipolar correlations of H_{18} oleate (methyl, δ = 0.77 - 0.90 ppm) in (a) OH_2 black group (color: **9** blue; **10** red; **11-12** green), (b) OH_3 blue group (**1** blue, **2** red, **5** green and **6** purple) and (c) OH_6 red group (**3** blue; **4** red; **7** green; **8** purple). **Figure S15.** DOSY experiments (600 MHz, 298 K) of isomer **7** performed in **(a)** D_2O, concentration of **7** increases by a factor 2 from green to blue contour plots; **(b)** DMSO, concentration of **7** increases by a factor 10 from green to blue contour plots. **Table S1**. Comparison of CCS directly measured with DTIMS with our CCS obtained after calibration of TWIMS. **Table S2.** Assigning the H1 hypothesis from the score obtained with Equation (S1).

Copyright: © 2022 by the authors. Licensee MDPI, Basel, Switzerland. This article is an open access article distributed under the terms and conditions of the Creative Commons Attribution (CC BY) license (https://creativecommons.org/licenses/by/4.0/).

(a)

(b)

(c)

(d)

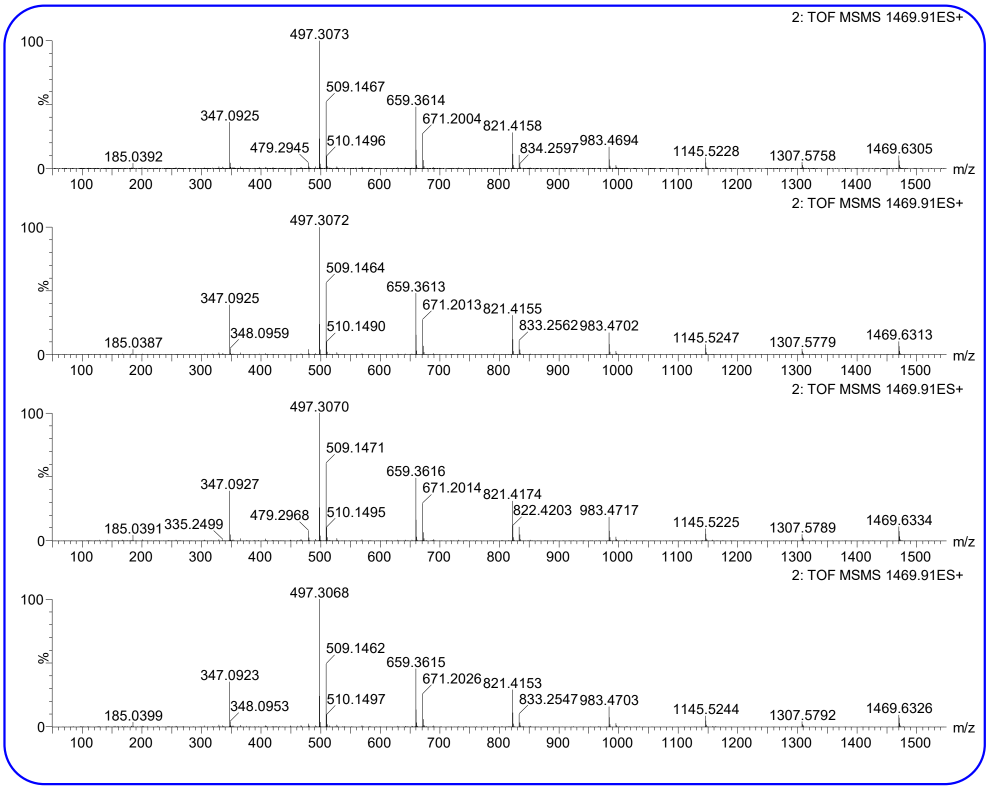

Figure S1. MS/MS spectra of [LipβCD+Na]+ (m/z 1469.63, EC = 95 eV) corresponding to the blue group isomers : **1 (a)**, **2 (b)**, **5 (c)** and **6 (d)**.

(a)

(b)

(c)

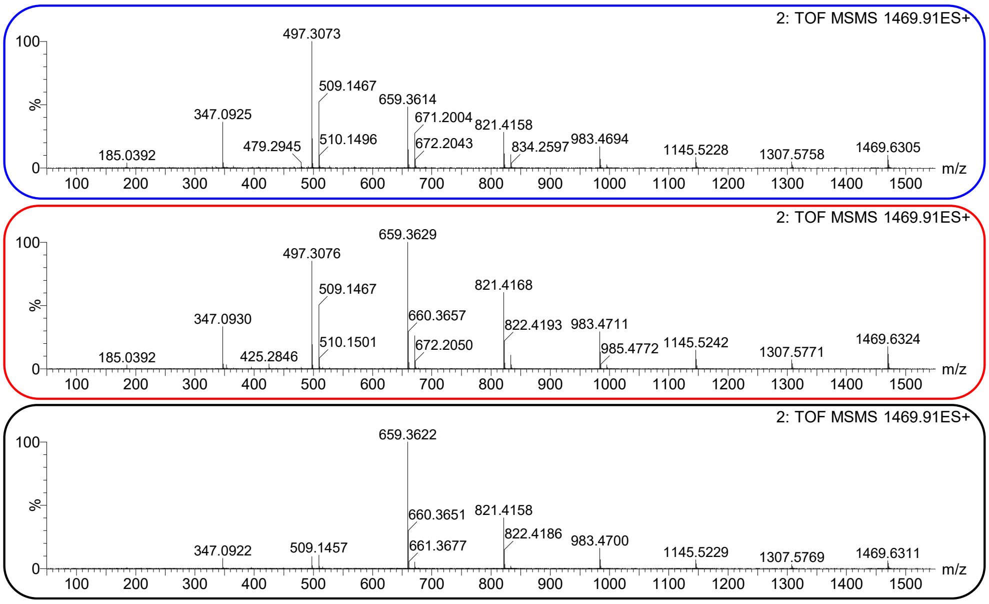

Figure S2. MS/MS spectra of [LipβCD+Na]$^+$ (m/z 1469.63, EC = 95 eV) corresponding to one isomer of each group: blue group **1** **(a)**, red group **3** **(b)** and black group **9** **(c)**. Note that spectra are identical for **(a)** compounds **1**, **2**, **5**, **6**; **(b)** compounds **3**, **4**, **7** and **8**; **(c)** compounds **9**, **10**, **11** and **12**.

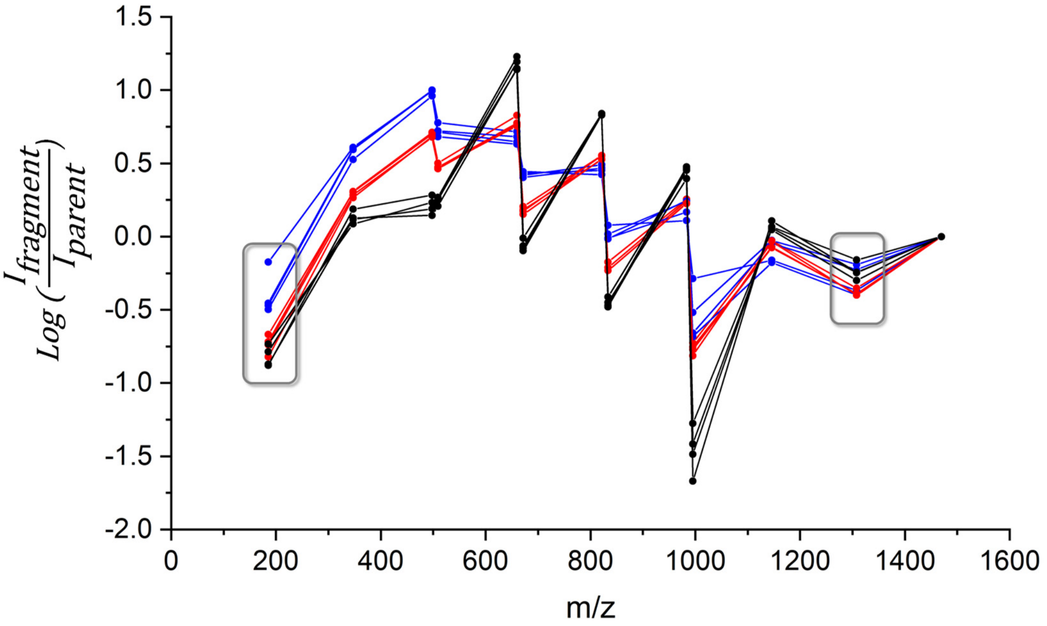

Figure S3. Raw data (points) used to build the PCA, obtained from the intensities of fragment ions observed in MS/MS spectra. Lines have been added to improve the readability of the differences between groups, represented by the colors blue, red and black. The boxes show the only two variables whose values in the red group are less than or equal to those in the blue and black groups.

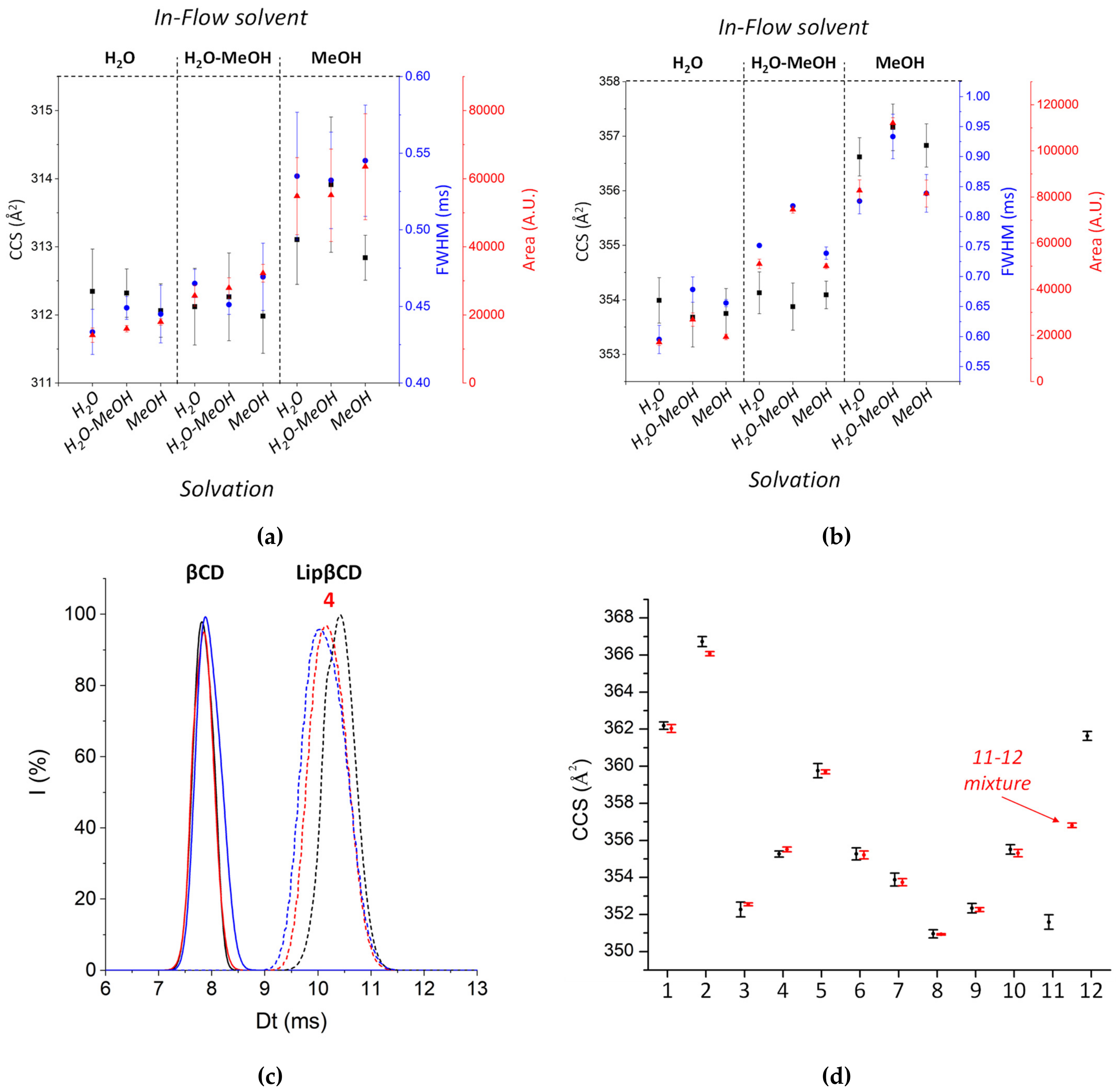

Figure S4. The descriptive values CCS (black), FWHM (blue) and area (red) of ion mobility distributions obtained with different solvents conditions (in-flow solvent and sample solvation) for native βCD **(a)** and LipβCD(OH_6) isomer **4** **(b)**. **(c)** Superimposition of the arrival time distributions obtained by the in-flow injection analysis (FIA) of pure native βCD and LipβCD(OH_6) isomer **4**, diluted in H_2O/MeOH 50/50 *v*/*v* and push with H_2O (blue), H_2O/MeOH 50/50 *v*/*v* (red) and MeOH (black) as in-flow solvent. **(d)** Comparison of CCS values obtained by UHPLC-IM-MS of the mixture (black) and by FIA-IM-MS of each pure isomers with H_2O/MeOH 50/50 *v*/*v* as in-flow solvent (red).

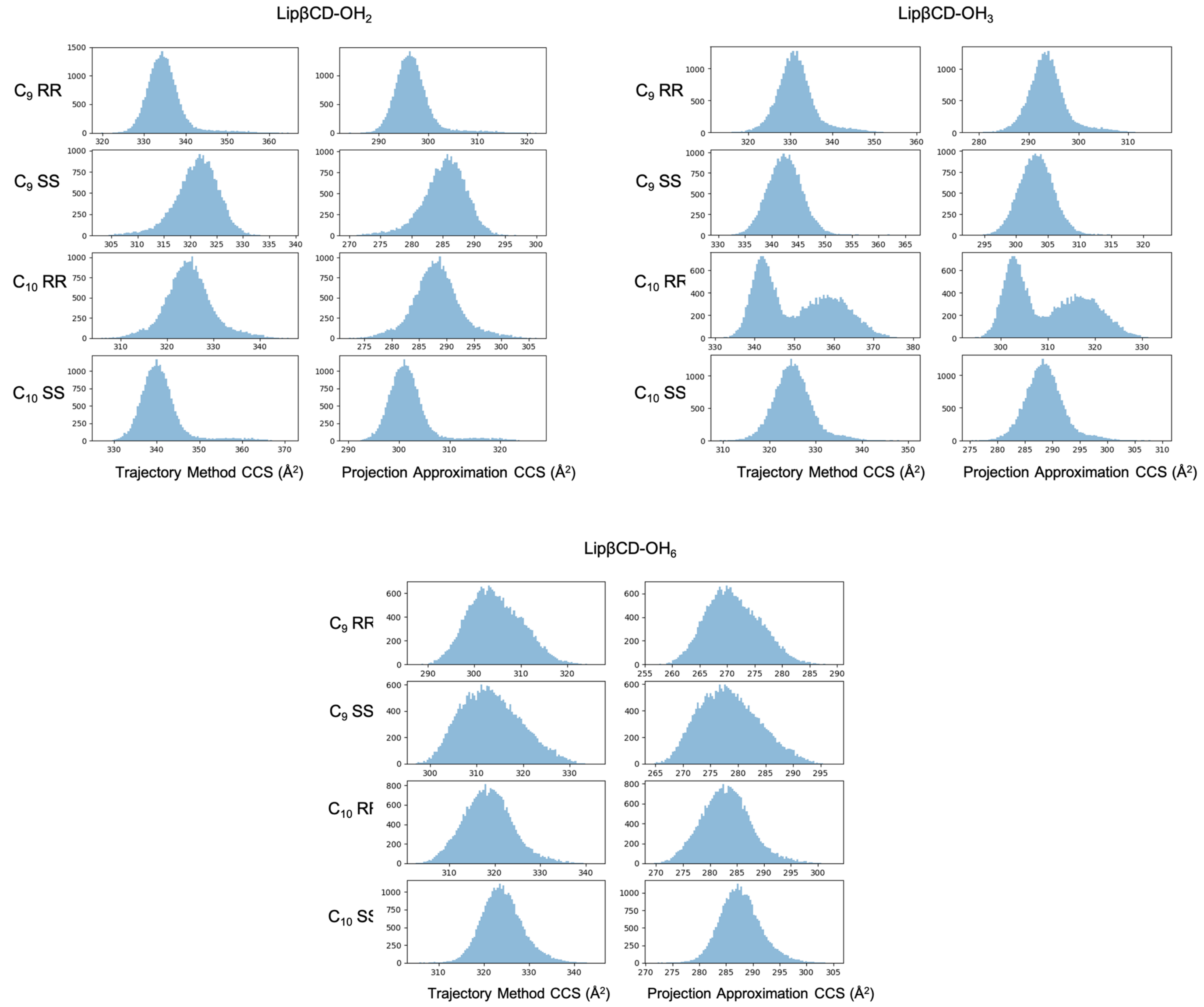

Figure S5. Distribution of theoretical CCS_{th} values obtained for the minimal structures of the twelve isomers of LipβCD(OH_2) using the Trajectory Method and the Projection Approximation.

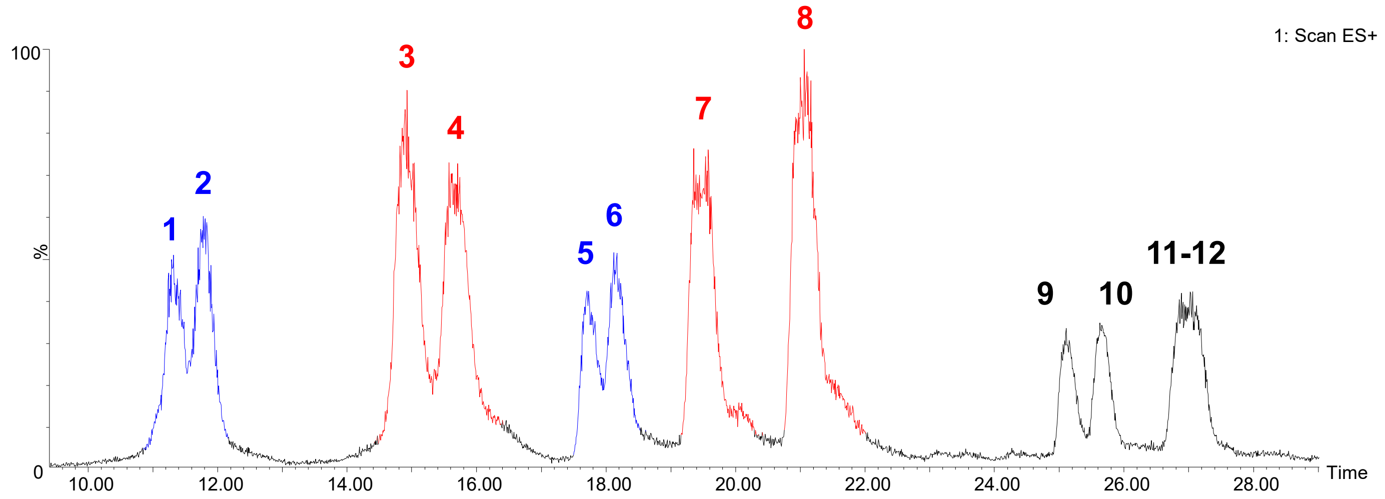

Figure S6. EIC of [LipβCD+Na]+ (m/z 1469) obtained from preparative scale LC-MS.

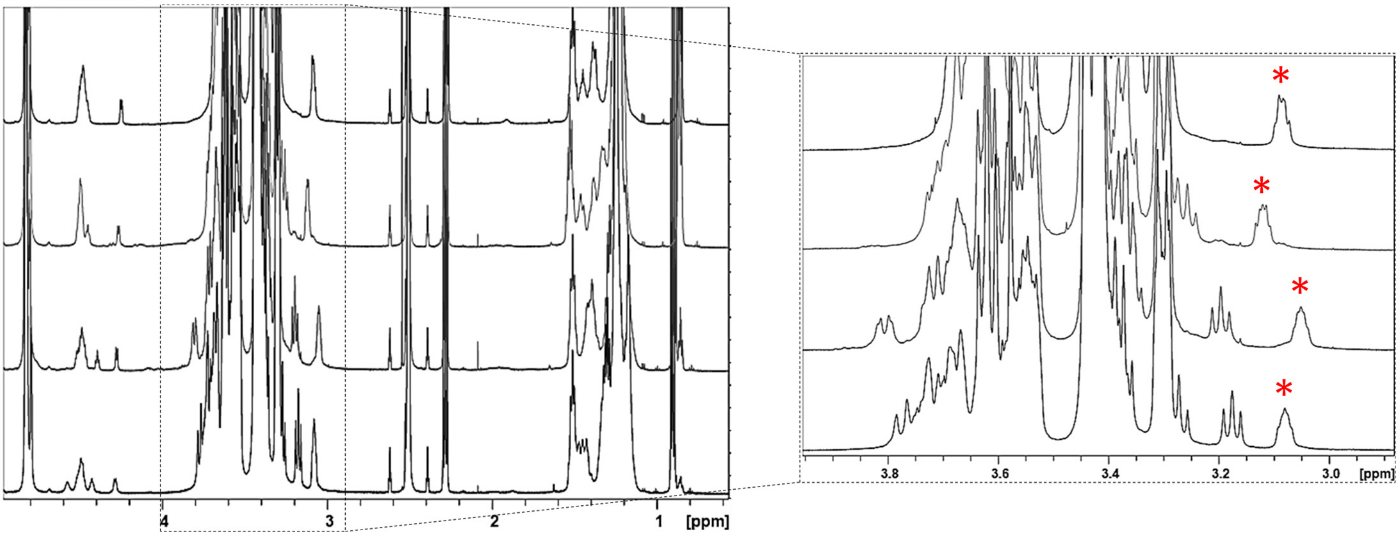

Figure S7. ^{1}H NMR spectra (600 MHz, DMSO/D_2O 98/2, *v*/*v*, 298 K) of isomers **3**, **4**, **7** and **8** (from bottom to top). The expansion of the spectral area (2.9 - 3.9 ppm) highlights the similarity of the spectra in the same group (red group here). The stars indicate the proton of C_{oleate} grafted on βCD.

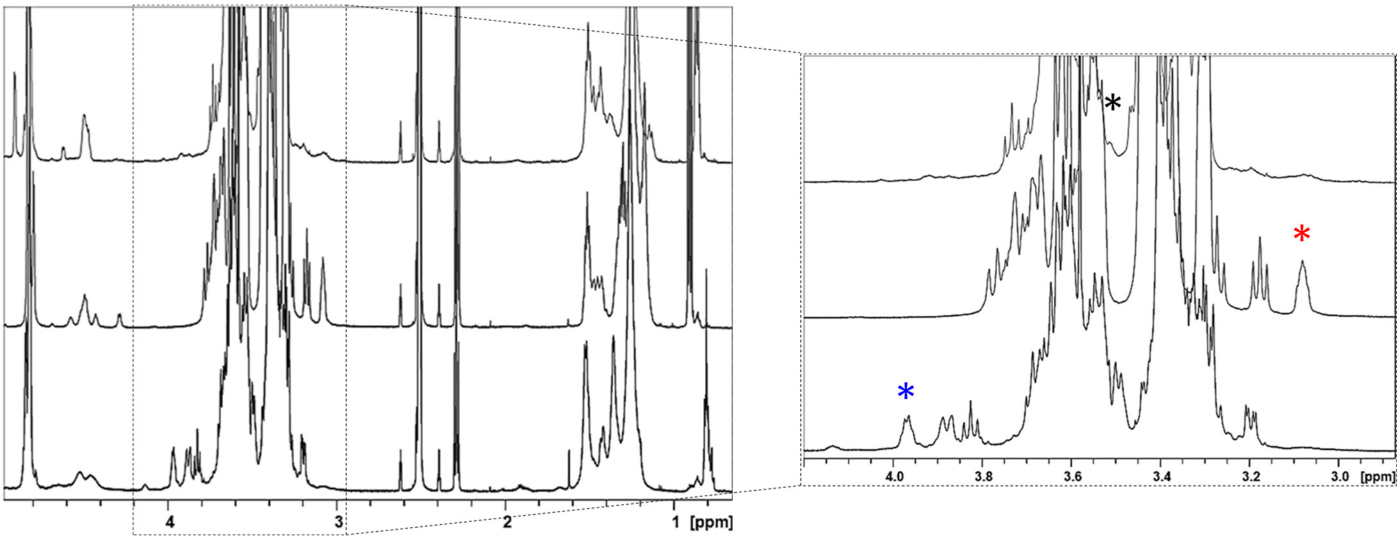

Figure S8. ^{1}H NMR spectra (600 MHz, DMSO/D_2O 98/2, *v*/*v*, 298 K) of isomers **1**, **3** and **9** (from bottom to top). The expansion of the spectral area (2.9 – 4.2 ppm) highlights the spectra differences between the groups. The stars indicate the proton of C_{oleate} grafted on βCD and the colors their corresponding group.

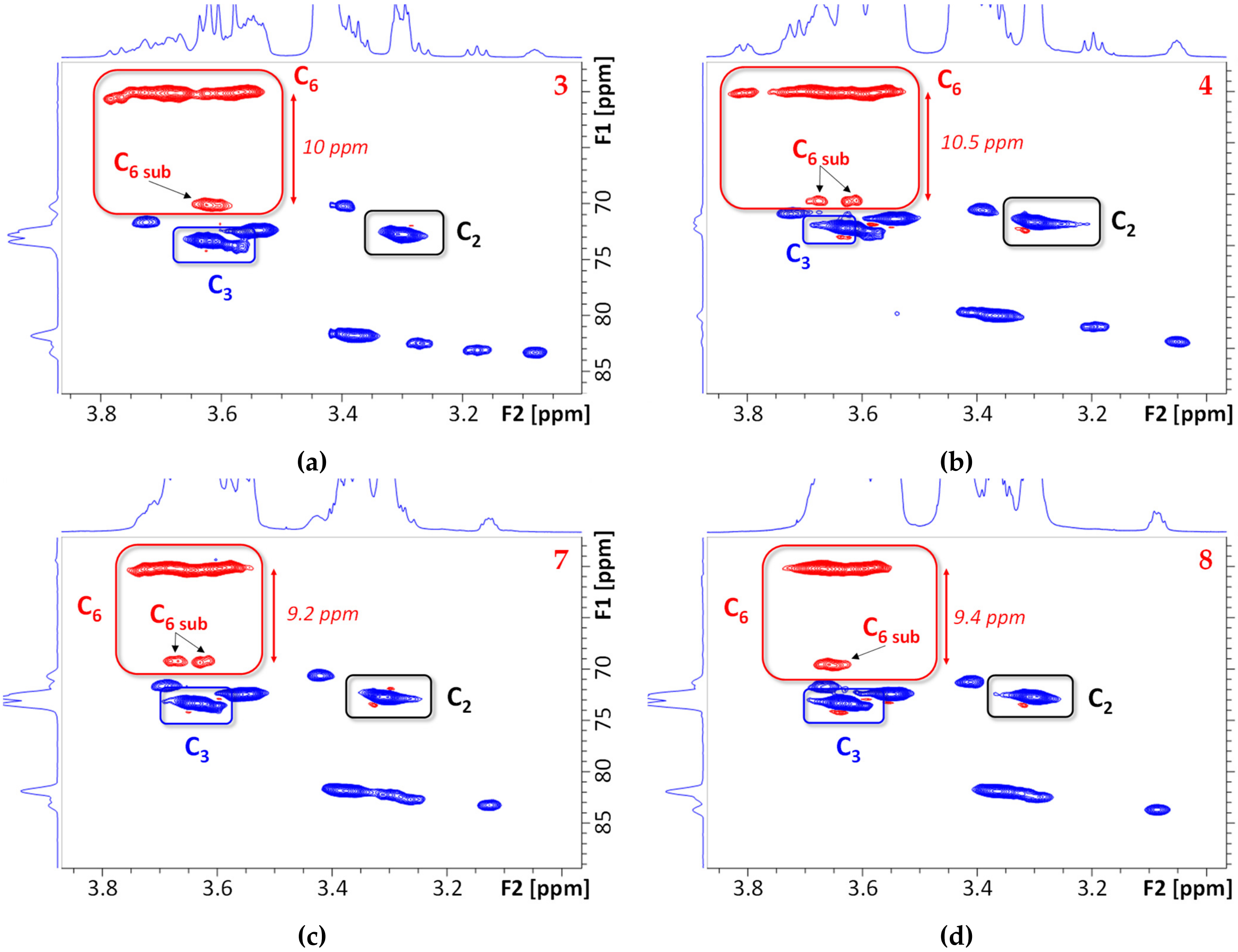

Figure S9. Partial contour plot of HSQC experiments (600 MHz, DMSO/D_2O 98/2, *v*/*v*, 298 K) of isomers **3** **(a)**, **4** **(b)**, **7** **(c)** and **8** **(d)** of the same LipβCD(OH_6) red group.

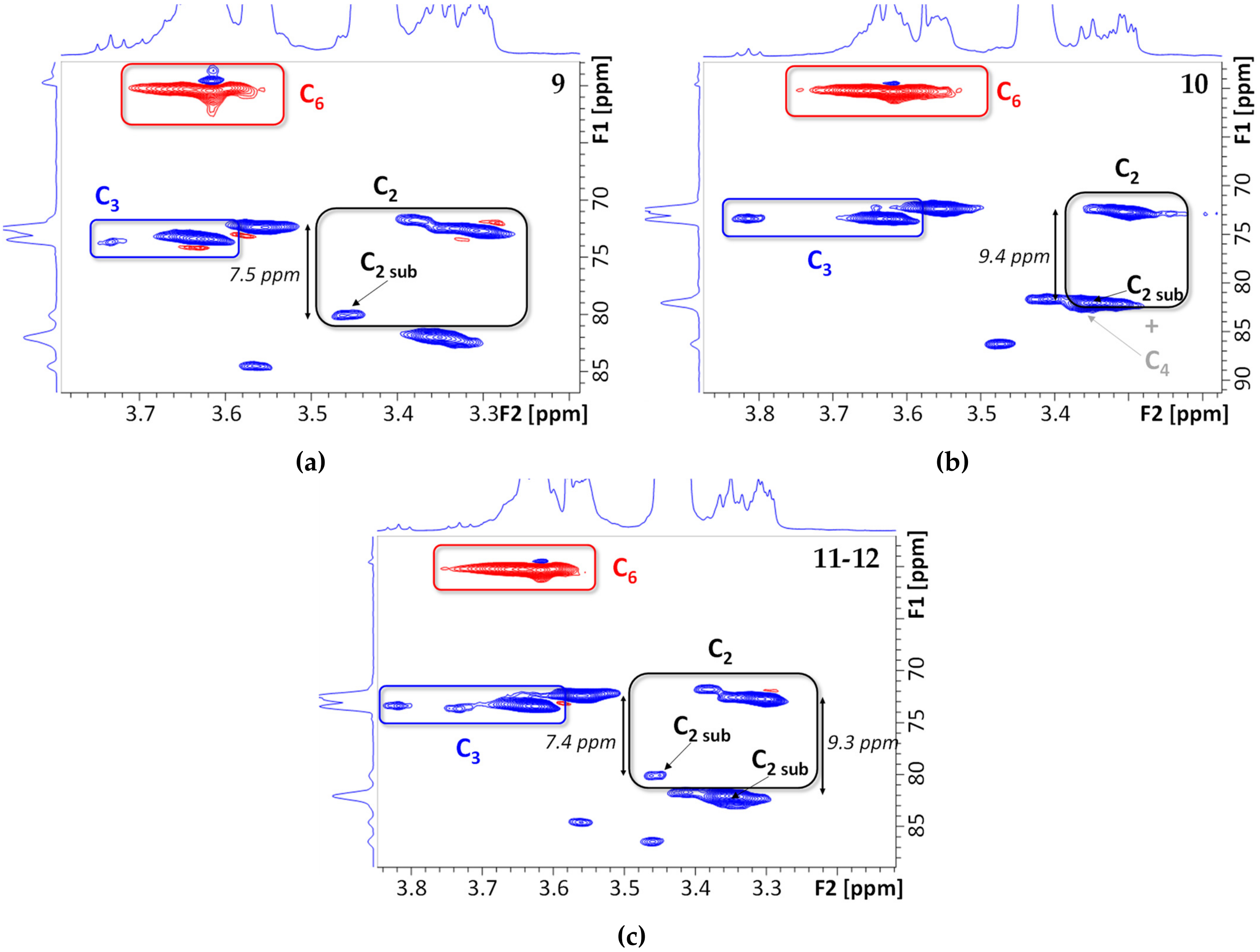

Figure S10. Partial contour plot of HSQC experiments (600 MHz, DMSO/D_2O 98/2, *v*/*v*, 298 K) of isomers **9 (a)**, **10 (b)** and **11-12 (c)** of the same LipβCD(OH_2) black group.

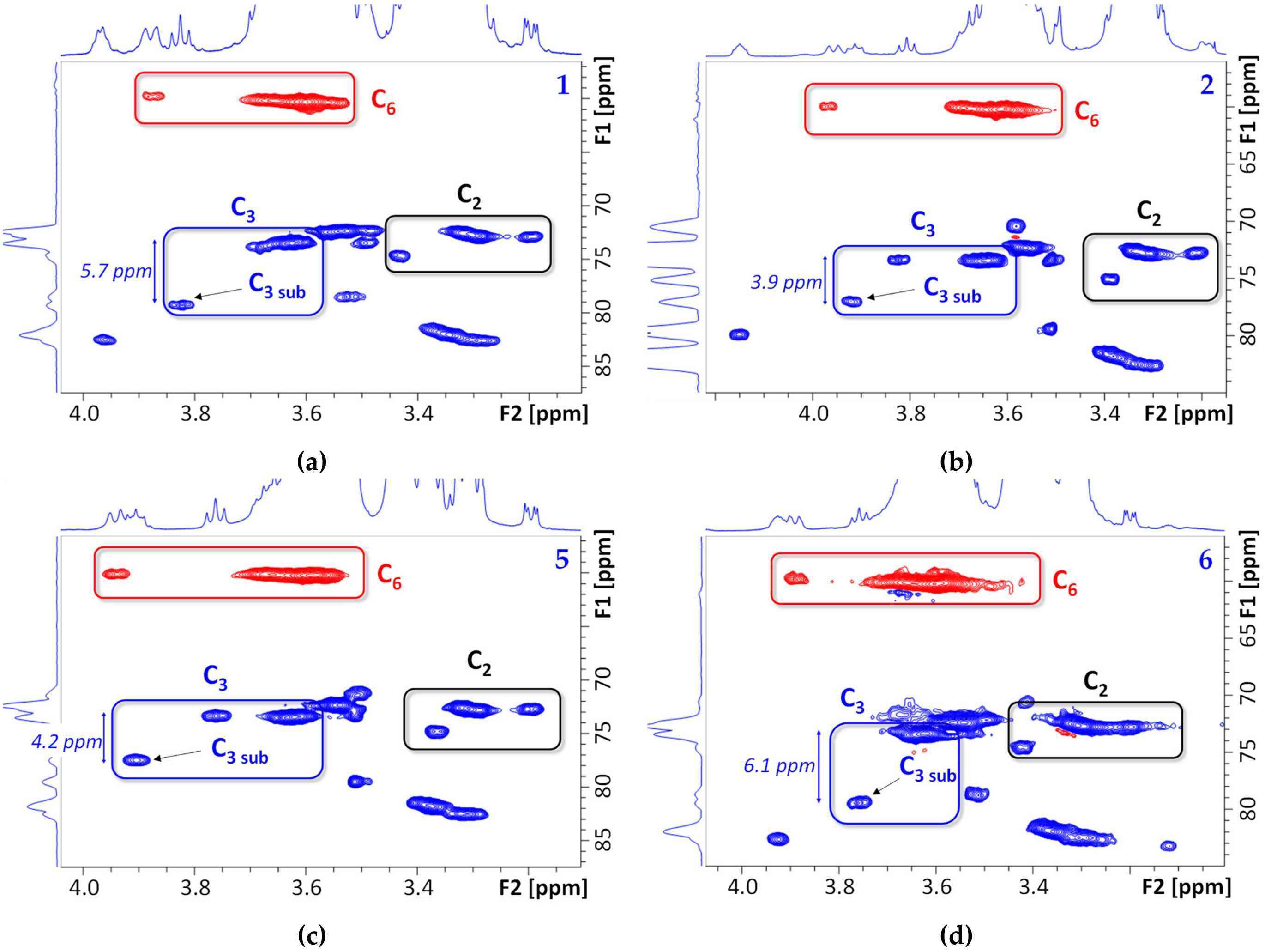

Figure S11. Partial contour plot of HSQC experiments (600 MHz, DMSO/D_2O 98/2, *v*/*v*, 298 K) of isomers **1 (a)**, **2 (b)**, **5 (c)** and **6 (d)** of the same LipβCD(OH_3) blue group.

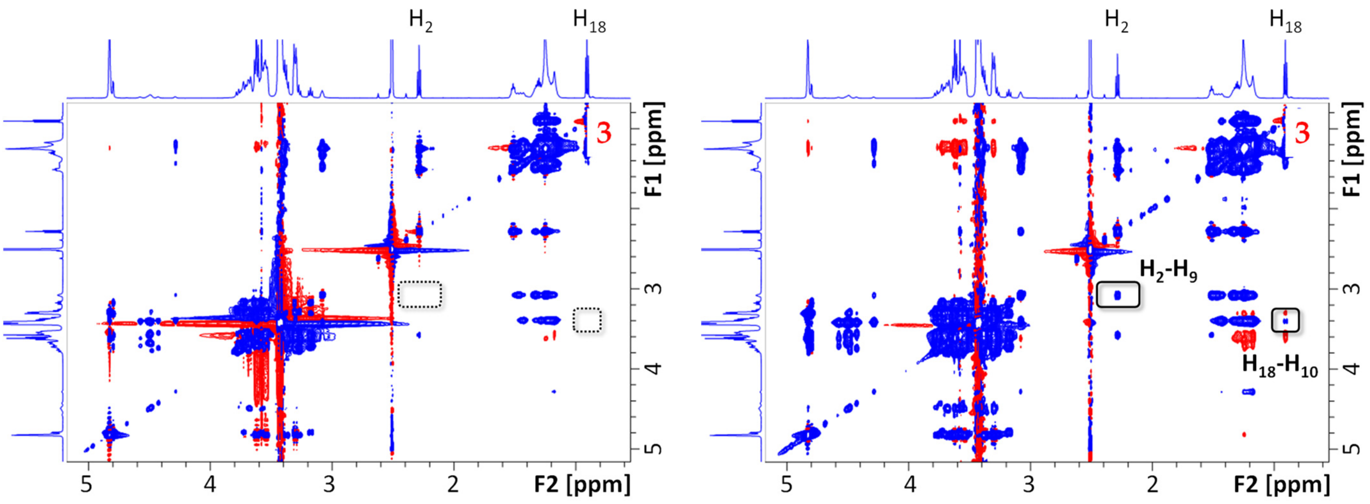

(a) **(b)**

Figure S12. 2D TOCSY experiments (600 MHz, DMSO/D_2O 98/2, *v/v*, 298 K) of isomer **3** performed with a mixing time of 100 ms **(a)** or 160 ms **(b)**. The presence or absence of long distance correlation peaks is highlighted by solid or dash rectangles.

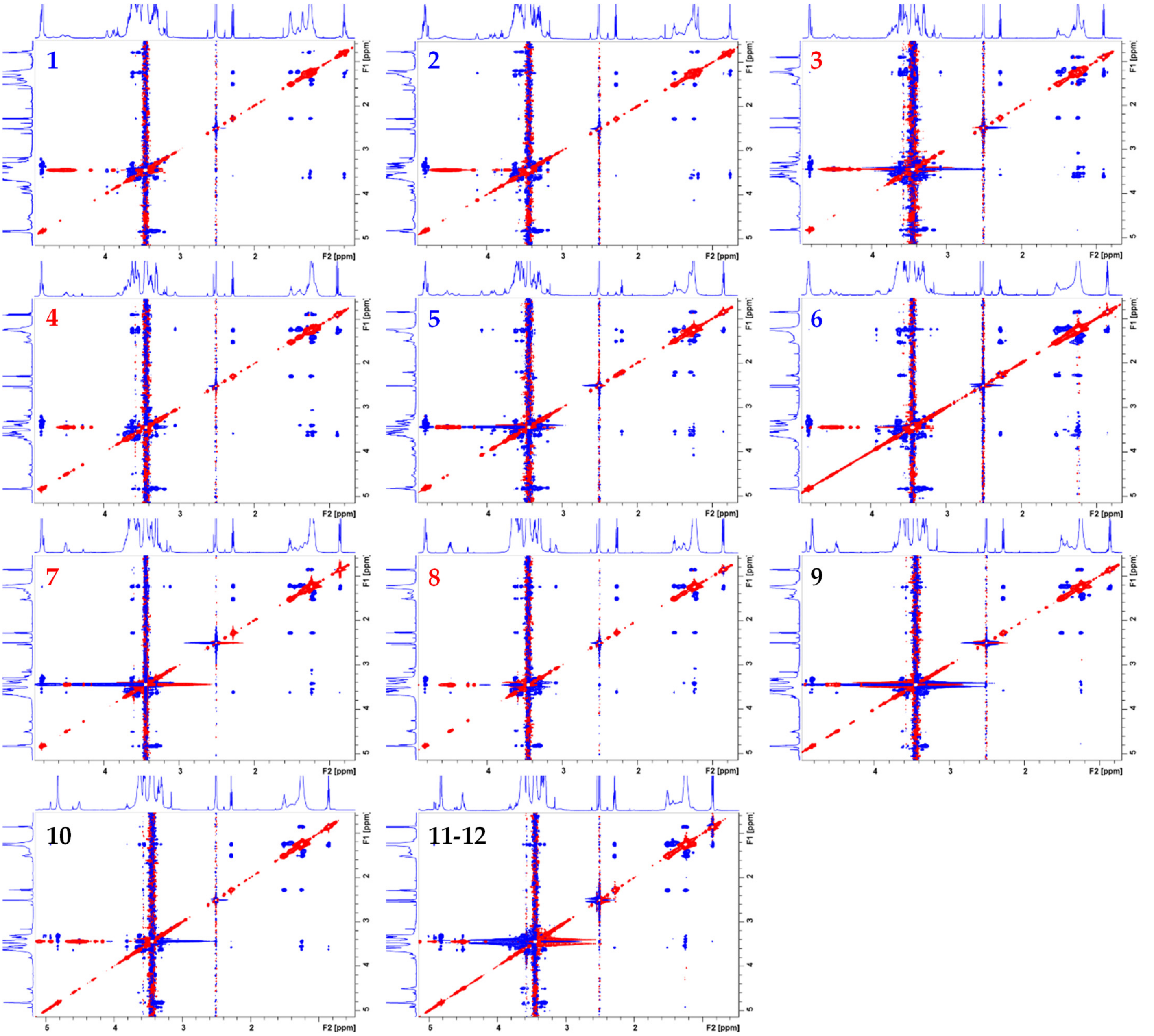

Figure S13. 2D ROESY experiments (600 MHz, DMSO/D$_2$O, 298 K 98/2, *v*/*v*, tm = 800 ms) of isomers **1- 12**.

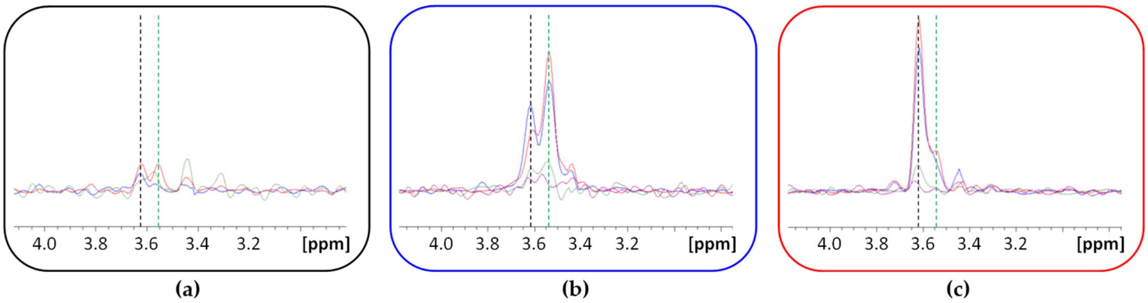

Figure S14. Superimposition of extracted columns from ROESY NMR experiments (600 MHz, DMSO/D_2O 98/2, *v/v*, 298 K, tm = 800 ms) containing dipolar correlations of $H_{18\ oleate}$ (methyl, δ = 0.77 - 0.90 ppm) in **(a)** OH_2 black group (color: **9** blue; **10** red; **11-12** green), **(b)** OH_3 blue group (**1** blue, **2** red, **5** green and **6** purple) and **(c)** OH_6 red group (**3** blue; **4** red; **7** green; **8** purple).

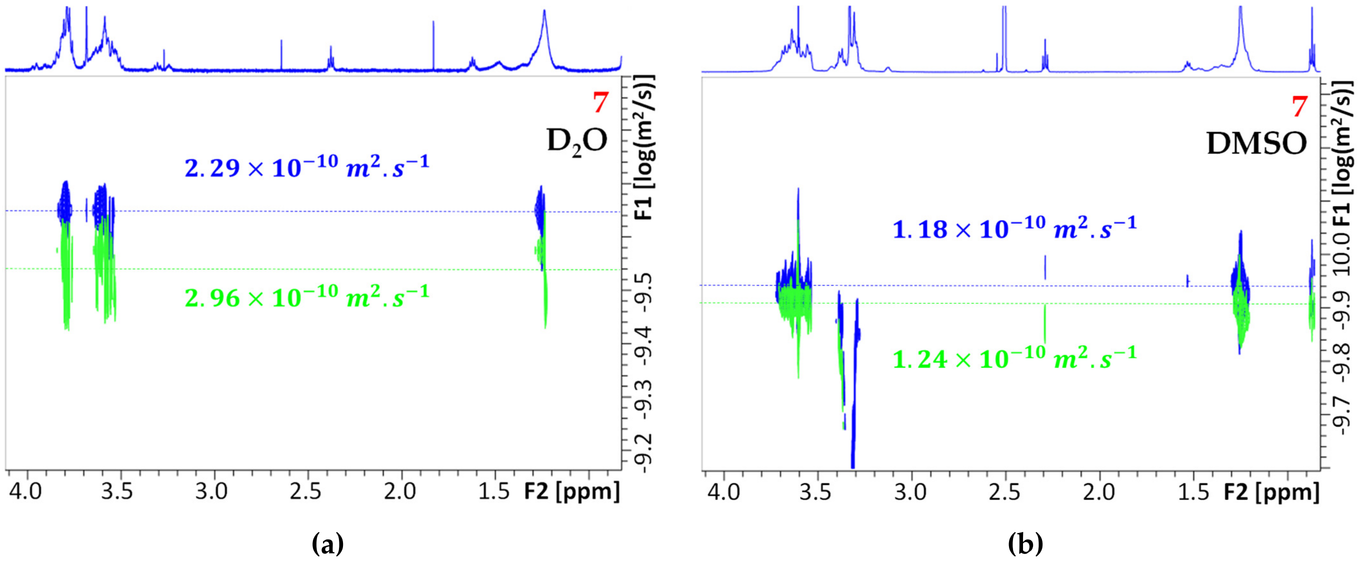

Figure S15. DOSY experiments (600 MHz, 298 K) of isomer **7** performed in **(a)** D_2O, concentration of **7** increases by a factor 2 from green to blue contour plots; **(b)** DMSO, concentration of **7** increases by a factor 10 from green to blue contour plots.

Table S1. Comparison of CCS directly measured by DTIMS* with our CCS obtained by TWIMS after calibration.

	CCS (DTIMS)*	CCS_{exp} (TWIMS)	Δ (%)
αCD	278.2	280.7	0.89
βCD	309.0	312.4	1.11
γCD	316.0	321.0	1.57

* Values obtained from Klein, C. et al. Analyst 2018, 143, 4147–4154. (Ref [32] in main text)

Table S2. Assigning the H1 hypothesis from the score obtained with Equation (S1).

	Experimental			Ranking		Theoretical			
**Isomer**	**CCS_{exp}**	**H1**	**Weight factor***	**$n_{i\,exp}$**	$n_{i\,th}$	Isomer LipβCD	$\overline{CCS_{th}}$	x_i	Score
**2**	366.36 ± 0.20	OH_3	11.35	1-OH_3	1-OH_3	(OH_3,C_9), SS	350.87	1	11.35
**1**	361.77 ± 0.14	OH_3	6.76	2-OH_3	2-OH_3	(OH_3,C_{10}), RR	342.42	1	6.76
**12**	361.24 ± 0.22	OH_2	6.24	3-OH_2	3-OH_2	(OH_2,C_{10}), SS	339.9	1	6.24
**5**	359.31 ± 0.21	OH_3	4.31	4-OH_3	4-OH_2	(OH_2,C_9), RR	334.24	0.5	2.15
**6**	355.05 ± 0.28	OH_3	0.05	5-OH_3	5-OH_3	(OH_3,C_9), RR	330.8	1	0.05
**10**	355.02 ± 0.25	OH_2	0.02	6-OH_2	6-OH_3	(OH_3,C_{10}), SS	324.81	0.5	0.01
**4**	354.99 ± 0.19	OH_6	0.02	7-OH_6	7-OH_2	(OH_2,C_9), SS	324.49	0.5	0.01
**7**	353.59 ± 0.27	OH_6	1.42	8-OH_6	8-OH_6	(OH_6,C_{10}), SS	323.7	1	1.42
**3**	352.23 ± 0.22	OH_6	2.77	9-OH_6	9-OH_2	(OH_2,C_{10}), RR	321.73	0.5	1.39
**9**	352.04 ± 0.16	OH_2	2.96	10-OH_2	10-OH_6	(OH_6,C_9), SS	318.33	0.5	1.48
**11**	351.28 ± 0.32	OH_2	3.73	11-OH_2	11-OH_6	(OH_6,C_{10}), RR	313.04	0.25	0.93
**8**	350.62 ± 0.19	OH_6	4.39	12-OH_6	12-OH_6	(OH_6,C_9), RR	304.41	1	4.39
		Sum	44.00						36.17

$*Weight\ factor = |Median_{CCSexp} - CCS_{exp}|$

As the dispersion of the CCS_{exp} is an important differentiating factor of the groups, we used the deviation from the median to weight the result, which led to the scoring system according to Equation (S1).

$$Score = \frac{\sum_{i=1}^{12} x_i \times |Median_{CCSexp} - CCS_{\exp i}|}{\sum_{i=1}^{12} |Median_{CCSexp} - CCS_{\exp i}|} \quad (S1)$$

With x_i = 1, 0.5 or 0.25 if $n_{i\,exp} = n_{i\,th}$, $n_{i\,th} \pm 1$ or $n_{i\,th} \pm 2$, respectively. x_i is a freedom factor and n_i the ranking place labelled with the grafted position on βCD. For clarity, the parameters of the equation are detailed for hypothesis H1 in Table 2. The maximum score is obtained for a perfect match of grafting position ($n_{i\,exp} = n_{i\,th}$, x_i = 1, e.g. isomer **2**). This score is divided by 2 when $n_{i\,exp}$ equals to $n_{i\,th} \pm 1$ (e.g. isomer **5,** $n_{i\,exp}$ = 4 and $n_{i\,th}$ = 5, x_i = 0.5) or by 4 ($n_{i\,exp} = n_{i\,th} \pm 2$, x_i = 0.25, e.g. isomer **11**) when two identical labels are shifted by one or two lines respectively. From the weighted comparison of $n_{i\,exp}$ with $n_{i\,th}$ for the six hypotheses, H1 hypothesis, exhibiting a score value of 82% (36.17/44.00 see on Table 2), is unambiguously the most consistent with the theoretical data: blue, red and black groups are corresponding respectively to the grafting position on OH_3, OH_6 and OH_2 of βCD.
